# Regulatory T cells in perioperative neurocognitive disorders: a systematic review with structured narrative synthesis from molecular mechanisms to clinical translation

**DOI:** 10.3389/fnmol.2026.1856316

**Published:** 2026-07-16

**Authors:** Yude Zhu, Ke Chai, Chenxu Chou, Xiaoyu Zhang, Yulong Jia, Xiaojun Ge, Xiaguang Duan

**Affiliations:** 1 Department of Interventional Neurology, Sinopharm North Hospital, Baotou, China; 2 Department of Pathology, South China Hospital, Medical School, Shenzhen University, Shenzhen, China; 3 Department of Anesthesiology, Inner Mongolia Baogang Hospital, Baotou, China; 4 Department of Anesthesiology, Sinopharm North Hospital, Baotou, China

**Keywords:** blood brain barrier, immune modulation, neuroinflammation, perioperative neurocognitive disorders, regulatory T cells

## Abstract

**Background:**

Perioperative neurocognitive disorders (PND), encompassing postoperative delirium and postoperative cognitive dysfunction, are common complications following anesthesia and surgery, particularly in elderly patients. Neuroinflammation has been identified as a central mechanism underlying PND; however, the contribution of adaptive immune regulation remains incompletely understood. Regulatory T cells (Tregs), as key modulators of immune homeostasis, have recently emerged as potential regulators of perioperative neuroinflammatory responses.

**Objective:**

This review aimed to comprehensively evaluate current preclinical and clinical evidence regarding the role of regulatory T cells in the development of perioperative neurocognitive disorders, with a focus on molecular mechanisms and translational implications.

**Methods:**

A systematic literature search was conducted in PubMed, PubMed Central, Web of Science, Scopus, Embase, the Cochrane Library, and ClinicalTrials.gov from inception to 17 May 2026. Original animal and human studies investigating Treg frequency, function, or modulation in relation to perioperative cognitive outcomes were included. Study selection followed PRISMA 2020 guidelines, and methodological quality was assessed using the SYRCLE risk-of-bias tool (animal studies) and the Newcastle–Ottawa Scale (clinical studies).

**Results:**

The search identified 312 records, of which 10 studies met the inclusion criteria, comprising four animal studies and six clinical studies. Preclinical evidence consistently demonstrated that surgical trauma induces alterations in Treg frequency and suppressive function, particularly in aged models, contributing to exaggerated neuroinflammation, blood–brain barrier disruption, microglial activation, and cognitive impairment. Clinical studies revealed associations between perioperative Treg imbalance—often reflected by an increased Th17/Treg ratio—and the occurrence of postoperative cognitive decline. Moreover, immunomodulatory interventions such as dexmedetomidine were shown to partially preserve Treg balance and reduce the incidence of postoperative cognitive dysfunction, although dexmedetomidine’s pleiotropic actions (sympatholysis, opioid-sparing, sedation-related stress reduction) preclude attribution of its cognitive benefit solely to Treg modulation.

**Conclusion:**

Current evidence supports a critical role for regulatory T cells in the pathophysiology of perioperative neurocognitive disorders. Treg dysfunction contributes to excessive neuroinflammation and cognitive decline after surgery, whereas restoration of Treg-mediated immune regulation confers neuroprotective effects. Targeting regulatory T cells represents a promising translational strategy for the prevention and management of PND, warranting further mechanistic studies and well-designed clinical trials.

## 1 Introduction

Perioperative neurocognitive disorders (PND) comprise a spectrum of cognitive disturbances temporally associated with surgery and anesthesia, including acute postoperative delirium (POD) and longer-lasting postoperative neurocognitive decline as formalized by recent international consensus (Shen et al., 2020; Borchers et al., 2024; Wang J. L. et al., 2025). The transition from the historically heterogeneous concept of postoperative cognitive dysfunction (POCD) to the unified PND framework aimed to standardize diagnostic timing and terminology, thereby improving comparability across studies (Liu L. et al., 2022; Muscat et al., 2023). However, despite this conceptual consolidation, perioperative cognitive impairment remains highly prevalent, particularly among older adults, and continues to exert profound consequences on postoperative recovery, long-term independence, and health-care utilization (Mahanna-Gabrielli et al., 2019; Suraarunsumrit et al., 2022; Berger et al., 2023; Travers et al., 2024). These observations suggest that terminological harmonization alone has not resolved the fundamental biological uncertainties underlying PND (Villa et al., 2025).

Neuroinflammation has emerged as a central mechanistic theme linking peripheral surgical stress to postoperative cognitive vulnerability (Saxena et al., 2021b; Bhuiyan et al., 2023; Koh and Joo, 2025). Surgical injury provokes systemic immune activation, disrupts blood–brain barrier integrity, and induces glial and inflammatory responses within cognition-related brain regions (Miller-Rhodes et al., 2022; Koh and Joo, 2025). Yet, mounting evidence indicates that neuroinflammation in PND is not merely a localized central phenomenon but reflects a broader failure of immune regulation across the perioperative period (Verdonk et al., 2024; Shi et al., 2025). Crucially, it remains unclear which regulatory pathways determine whether postoperative inflammation is effectively resolved or instead evolves into sustained neurocognitive dysfunction (Dillon et al., 2023).

Regulatory T cells (Tregs), defined by expression of CD4, CD25, and the transcription factor FOXP3, are pivotal mediators of immune homeostasis and inflammatory resolution (Jovisic et al., 2023; Kain et al., 2024; Meng T. et al., 2025). Beyond their canonical roles in autoimmunity and transplantation, Tregs have been increasingly implicated in neuroimmune regulation across a range of neurological conditions (Choi et al., 2022; Harkins et al., 2022; Gao et al., 2023). In perioperative models, alterations in Treg abundance or function—and in the balance between Tregs and pro-inflammatory T helper 17 (Th17) cells—have been associated with exaggerated neuroinflammatory responses and impaired cognitive outcomes (Verdonk et al., 2024; Ying and Lin, 2025). Nevertheless, the role of Tregs in PND remains conceptually unsettled. Experimental studies differ in their temporal focus, anatomical compartment of interest, and outcome definitions, while clinical evidence is limited and often inconsistent (Huang et al., 2025). Whether Tregs primarily act as peripheral immune gatekeepers, direct modulators of central neuroinflammation, or dynamic integrators of systemic and neural immune responses remains unresolved (Dou et al., 2023; Zhao Y. et al., 2025).

These uncertainties point to a critical conceptual gap in the current understanding of perioperative neurocognitive disorders. While neuroinflammation is widely accepted as a contributing mechanism, the immunoregulatory architecture governing its initiation, propagation, and resolution has not been systematically integrated across preclinical and clinical domains (Hsu et al., 2022; Hernandez et al., 2023; Moe et al., 2024). In particular, the position of Tregs within this architecture—and their relevance as potential modulators of postoperative cognitive trajectories—has yet to be coherently defined (Zhou et al., 2023).

In this systematic review with structured narrative synthesis, we synthesize evidence from experimental and clinical studies to delineate the role of regulatory T cells in perioperative neurocognitive disorders within the contemporary PND framework. By critically integrating heterogeneous findings, we aim to refine mechanistic understanding, expose sources of inconsistency, and propose a translationally relevant immunoregulatory model that may inform future perioperative research and intervention strategies.

## 2 Methods

### 2.1 Study design and reporting framework

This systematic review with structured narrative synthesis was conducted and reported in accordance with the Preferred Reporting Items for Systematic Reviews and Meta-Analyses (PRISMA) 2020 guidelines (Sequeira et al., 2025; Walizada et al., 2025; Laaksonen et al., 2026). We retain the “systematic review” label because the work fulfills the core PRISMA 2020 definitional criteria—namely, a pre-specified protocol, reproducible multi-database search strategy, dual independent screening, formal risk-of-bias assessment, and transparent synthesis. PRISMA 2020 explicitly accommodates systematic reviews that adopt narrative synthesis when quantitative pooling is precluded by methodological or clinical heterogeneity (PRISMA 2020 item 13d). A predefined review protocol was developed prior to literature screening to specify the search strategy, eligibility criteria, data extraction variables, and synthesis plan. Given the mechanistic focus of the research question and the anticipated heterogeneity across experimental models and clinical studies, we adopted a structured narrative synthesis framework designed to integrate preclinical and clinical evidence while preserving their distinct inferential roles. The review was not registered in PROSPERO; an internal protocol document is available from the corresponding author on reasonable request.

### 2.2 Literature search strategy

A comprehensive literature search was conducted across PubMed (MEDLINE), PubMed Central, Web of Science Core Collection, Scopus, Embase, the Cochrane Central Register of Controlled Trials (CENTRAL), and ClinicalTrials.gov from database inception to 17 May 2026. Database-specific search strategies were developed using a combination of controlled vocabulary (Medical Subject Headings [MeSH] in PubMed and Emtree terms in Embase) and free-text keywords related to regulatory T cells and perioperative neurocognitive disorders.

The primary search concepts comprised regulatory T cells (“regulatory T cell*,” “Treg*,” “FOXP3”) and perioperative cognitive outcomes (“perioperative neurocognitive disorder*,” “postoperative cognitive dysfunction,” “POCD,” “postoperative delirium,” “POD”). Search strategies were adapted to the syntax, indexing systems, and field tags of each database. No restrictions were placed on publication date. The full, reproducible search strategies for all databases, including search fields, Boolean operators, and date of execution, are provided in Supplementary File S1.

To minimize the risk of missing relevant studies, reference lists of all eligible articles and pertinent reviews were manually screened. ClinicalTrials.gov (Cambriel et al., 2026) was searched to identify completed or ongoing studies evaluating perioperative immune modulation and cognitive outcomes, enabling assessment of potential publication bias.

### 2.3 Eligibility criteria

Studies were eligible for inclusion if they met the following criteria:

*Study type*: Original experimental or clinical investigations.

*Population*: Animal models involving surgical procedures or adult patients undergoing surgery.

*Exposure or focus*: Quantitative or qualitative assessment of regulatory T cell frequency, phenotype, function, or experimental modulation in the perioperative context.

*Outcomes*: Evaluation of postoperative cognitive performance and/or neuroinflammatory endpoints relevant to perioperative neurocognitive disorders.

Studies were excluded if they were review articles, editorials, commentaries, or conference abstracts lacking primary data, or if they did not assess perioperative cognitive outcomes.

To address terminological heterogeneity, studies employing legacy definitions (e.g., postoperative cognitive dysfunction) were retained but interpreted within the contemporary perioperative neurocognitive disorder framework where applicable (Borchers et al., 2024; Sim et al., 2025).

### 2.4 Study selection

Two reviewers independently screened titles and abstracts for eligibility, followed by full-text assessment of potentially relevant articles. Disagreements at any stage were resolved through discussion and consensus. The study selection process is summarized in a PRISMA flow diagram presented in the Results section (Di Molfetta et al., 2022; Nizam et al., 2022).

### 2.5 Data extraction

Data were independently extracted by two reviewers using a predefined and piloted data extraction form. Extracted variables included study design, species or patient characteristics, type of surgical procedure or experimental model, timing of immune and cognitive assessments, methods used to quantify regulatory T cells, cognitive outcome measures, neuroinflammatory markers, and key mechanistic findings. Discrepancies were resolved by consensus.

### 2.6 Risk of bias assessment

Risk of bias in animal studies was assessed using the SYRCLE risk-of-bias tool (Wang et al., 2021; Cardoso et al., 2022). Clinical observational studies were evaluated using the Newcastle–Ottawa Scale (Purcell et al., 2022; Aquarius et al., 2023; Patel and Oremus, 2023). All assessments were performed independently by two reviewers, with disagreements resolved through discussion. Risk-of-bias assessments were considered during evidence synthesis to contextualize the strength and limitations of the included findings. Results of these assessments are reported in Section 3.4 and Tables 1 and 2. Certainty in the body of evidence for each major outcome was rated qualitatively, considering the small number of studies, the predominance of observational clinical designs, and consistency of findings across studies.

**Table 1:** SYRCLE risk of bias assessment for animal studies.

Study	Sequence generation	Allocation concealment	Blinding (caregivers)	Random housing	Blinding (outcome)	Incomplete outcome data	Selective reporting
Zhou et al. (2023)	L	U	U	L	L	L	L
Terrando et al. (2011)	U	U	U	L	U	L	L
Cibelli et al. (2010)	U	U	U	L	U	L	L
Vacas et al. (2014)	U	U	U	L	U	L	L
Safavynia and Goldstein (2018)	U	U	U	L	U	L	L

L = Low risk; U = Unclear risk; H = High risk. Domain judgments based on the SYRCLE risk-of-bias tool for animal studies (Hooijmans et al., 2014, BMC Med Res Methodol 14:43; 24667063). Two reviewers scored each domain independently; disagreements were resolved by consensus.

**Table 2:** Newcastle–Ottawa Scale assessment for clinical studies.

Study	Selection (max 4)	Comparability (max 2)	Outcome/Exposure (max 3)	Total (max 9)	Overall quality
Zhao et al. (2023b)	3	1	2	6	Moderate
Zhang et al. (2021)	3	1	2	6	Moderate
Wang J. et al. (2022)	3	2	2	7	Good
Wang et al. (2023)	3	1	2	6	Moderate
Verdonk et al. (2024)	4	2	2	8	Good
Tian et al. (2015)	3	2	2	7	Good
Hshieh et al. (2018) ^†^	3	1	1	5	Moderate (contextual)

^†^Hshieh et al. (2018) is a meta-analysis of the Hospital Elder Life Program (HELP) for delirium prevention; it does not directly assess Treg phenotype or function. During revision it was reclassified as contextual evidence and is no longer counted within the primary Treg-profiling synthesis pool, but is retained in this table for transparency. The Newcastle–Ottawa Scale was applied as described by Wells et al. (2014), (Ottawa Hospital Research Institute). Two reviewers scored each domain independently; disagreements were resolved by consensus.

### 2.7 Data synthesis

Owing to substantial heterogeneity in experimental models, immune assessment techniques, cognitive outcome measures, and timing of evaluations, quantitative meta-analysis was not undertaken (Nakamura et al., 2022). Instead, we conducted a structured narrative synthesis, stratifying evidence by study type (preclinical versus clinical), immune compartment (peripheral versus central), and perioperative time frame. This approach was chosen to enable mechanistic integration across heterogeneous studies while maintaining transparency regarding inferential boundaries (Kleinstreuer and Hartung, 2024).

## 3 Results

### 3.1 Study selection

**Figure 1:**
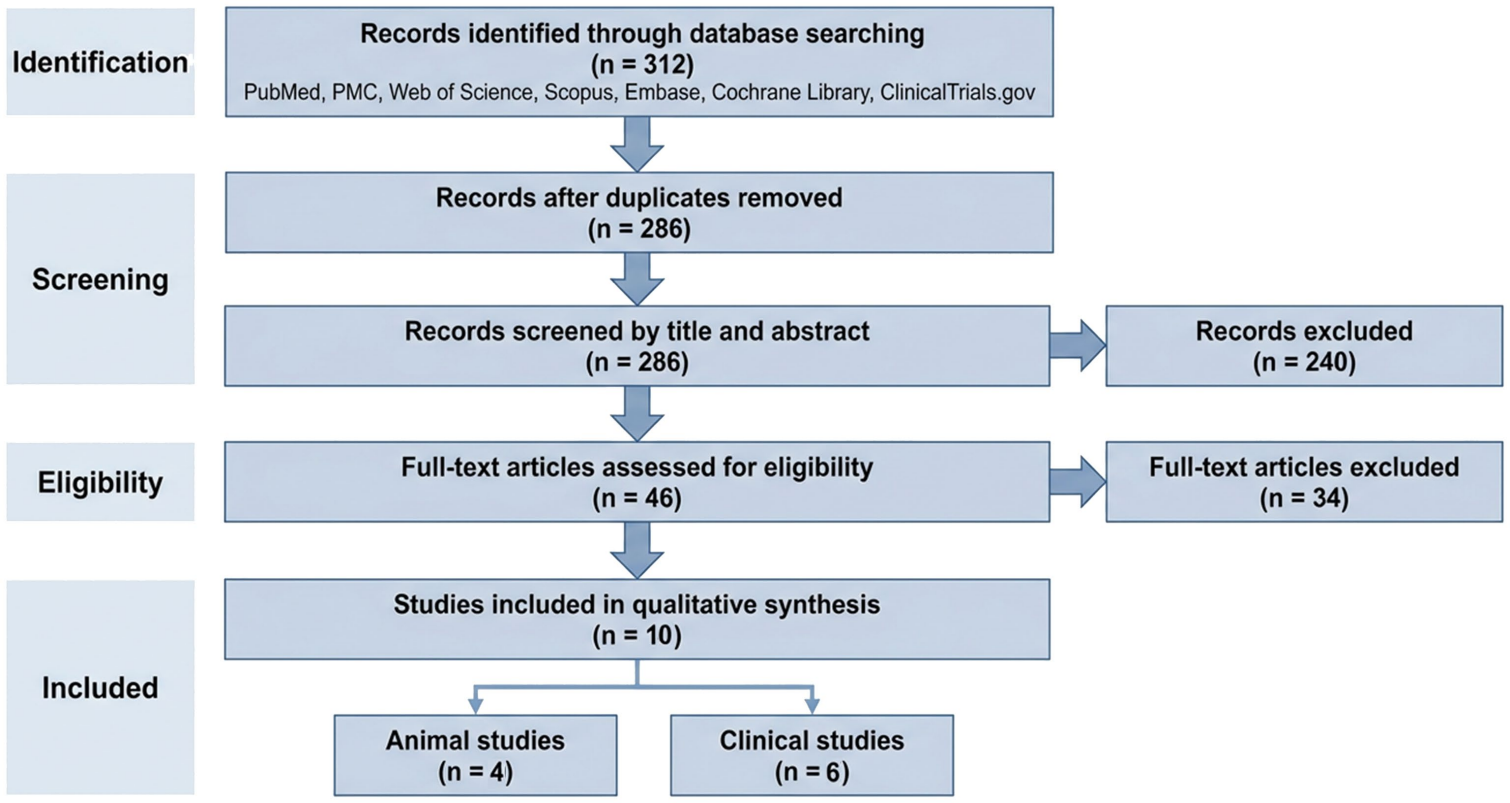
PRISMA flow diagram.

The search yielded 312 records. After duplicate removal and title/abstract screening, 46 articles were selected for full-text review. Of these, 10 studies met inclusion criteria for data synthesis: 4 animal studies and 6 clinical studies. A symmetric inclusion audit was applied to both pools: studies that did not directly assess Treg frequency, phenotype, or function were reclassified as contextual mechanistic evidence (denoted † in Tables 3, 4) but retained for narrative synthesis. The low inclusion-to-screening ratio reflects the stringent intersection of three required concepts (a perioperative surgical insult, direct Treg phenotypic or functional assessment, and a cognitive outcome relevant to PND). Most excluded full-text articles addressed only one or two of these elements; for example, studies of postoperative inflammation without Treg measurement, Treg studies without perioperative context, or PND studies without immune profiling. A detailed list of the 34 full-text articles excluded after eligibility assessment, with explicit reasons, is provided in Supplementary Table S1. The study selection process is depicted in the PRISMA flow diagram (Figure 1).

**Table 3:** Characteristics of included animal studies investigating regulatory T cells in perioperative neurocognitive disorders.

Author (year)	Animal model	Surgical/experimental procedure	Treg assessment method	Cognitive assessment	Main findings related to Tregs
Zhou et al. (2023)	Young and aged C57BL/6 mice	Tibial fracture fixation under anesthesia	Flow cytometry (CD4^+^CD25^+^FoxP3^+^), RNA sequencing	Morris water maze	Aged mice exhibited increased circulating Treg numbers with impaired suppressive function; adoptive transfer of young Tregs partially restored blood–brain barrier integrity and improved postoperative cognitive performance
Terrando et al. (2011)†	Adult mice	Laparotomy with tissue manipulation	† Cytokine profiling; no direct Treg quantification	Contextual fear conditioning	Peripheral immune activation following surgery promoted neuroinflammation and cognitive decline; adaptive immune regulation, including T cell–mediated mechanisms, contributed to postoperative cognitive impairment
Cibelli et al. (2010)†	Adult mice	Surgical trauma model	† IL-1β/inflammatory cytokine analysis; no direct Treg quantification	Behavioral memory tasks	Postoperative cognitive dysfunction was driven by exaggerated inflammatory responses; immune-modulating pathways involving T cell subsets influenced neuroinflammatory cascades
Vacas et al. (2014)†	Adult mice	Laparotomy	† HMGB1/bone-marrow-derived macrophage analysis; no direct Treg quantification	Trace fear conditioning	Surgery-induced systemic inflammation disrupted blood–brain barrier integrity and activated neuroinflammatory signaling; adaptive immune cells contributed to sustained cognitive deficits

^†^Reclassified during revision as contextual mechanistic evidence (no direct assessment of Treg frequency, phenotype, or function). Retained in table for transparency and narrative synthesis.

**Table 4:** Summary of clinical studies linking regulatory T cells to PND.

Author (year)	Study design	Patient population	Type of surgery	Immune parameters assessed	Cognitive outcomes	Main findings related to Tregs
Zhao et al. (2023a)	Randomized controlled trial	Elderly patients	Orthopedic surgery	Th17/Treg ratio (flow cytometry)	POCD incidence (neuropsychological testing)	Dexmedetomidine significantly reduced POCD incidence and restored perioperative Th17/Treg balance, suggesting a protective role of regulatory T cell–mediated immune modulation
Zhang X. et al. (2025)	Prospective observational study	Elderly surgical patients	Non-cardiac surgery	FoxP3^+^ Tregs, inflammatory cytokines	POCD diagnosis	Patients who developed POCD exhibited reduced postoperative FoxP3 expression and enhanced pro-inflammatory immune responses, indicating impaired regulatory T cell activity
Li et al. (2022)	Case–control study	Surgical patients	Cardiac surgery	Peripheral immune cell profiling	Postoperative delirium	Patients with postoperative delirium showed altered adaptive immune responses, including dysregulation of T cell subsets consistent with reduced immune regulation
Zhu et al. (2018)	Prospective observational study	Elderly patients	Major surgery	Circulating Tregs, cytokine levels	POCD at early postoperative period	Lower postoperative regulatory T cell levels were associated with higher systemic inflammation and increased POCD risk
Hshieh et al. (2018) ^†^	Prospective cohort study	Older hospitalized patients	Major surgery	Inflammatory and immune biomarkers	Postoperative delirium	Inflammatory immune signatures associated with delirium suggest a role for impaired immune regulation, potentially involving regulatory T cell pathways
Wang J. et al. (2022)	Observational study	Geriatric patients	Orthopedic surgery	Th17/Treg ratio, IL-6, TNF-α	POCD incidence	Exaggerated postoperative inflammation accompanied by Th17/Treg imbalance was associated with worse cognitive outcomes

^†^Reclassified during revision as contextual evidence (HELP delirium prevention trial; not a direct Treg-profiling study). Retained in table for transparency.

PRISMA flow diagram illustrating the literature search and study selection process for this systematic review. A total of 312 records were identified through database searching (PubMed, PubMed Central, Web of Science, Scopus, Embase, the Cochrane Library, and ClinicalTrials.gov). After removal of duplicates and screening of titles and abstracts, 46 full-text articles were assessed for eligibility. Ten studies met the inclusion criteria and were included in the qualitative synthesis, comprising four animal studies and six clinical studies.

### 3.2 Preclinical evidence: regulatory T cells in experimental PND

#### 3.2.1 Alterations in Treg dynamics after surgery

Accumulating preclinical evidence indicates that surgical trauma induces profound and dynamic alterations in regulatory T cell (Treg) homeostasis, encompassing changes in cell number, phenotype, and suppressive function (Handke et al., 2021). These perioperative Treg responses are highly context dependent and appear to be influenced by factors such as age, magnitude of surgical injury, and the inflammatory milieu.

In murine models of perioperative neurocognitive disorders, surgery triggers a rapid systemic inflammatory response characterized by elevated circulating pro-inflammatory cytokines, including tumor necrosis factor-*α* (TNF-α) and interleukin-6 (IL-6) (Wang J. et al., 2022; Dow and Kidess, 2025; Hao et al., 2025). In parallel, alterations in peripheral Treg populations have been consistently observed (Haunhorst et al., 2022; Wang W. et al., 2022). Notably, Zhou et al. demonstrated that aged mice subjected to tibial fracture fixation exhibited a significant postoperative increase in circulating CD4^+^CD25^+^FoxP3^+^ Tregs compared with young counterparts (Zhou et al., 2023). However, this numerical expansion was paradoxically accompanied by impaired suppressive function, suggesting that quantitative increases in Tregs do not necessarily reflect effective immune regulation in the perioperative setting (Zhang X. et al., 2025).

Functional impairment of postoperative Tregs was further supported by transcriptomic analyses, which revealed enrichment of inflammatory signaling pathways, including TNF signaling and cytokine–cytokine receptor interaction pathways, within Tregs isolated from aged surgical mice (Luo et al., 2021; Qin et al., 2024). These findings indicate that aging alters the molecular programming of Tregs, rendering them less capable of suppressing excessive inflammatory responses following surgery (Nemes et al., 2021; Šileikienė and Jurgauskienė, 2025). Consequently, dysfunctional Tregs may fail to restrain systemic inflammation, thereby amplifying downstream neuroinflammatory cascades (Faridar et al., 2022; Graber et al., 2025).

Beyond age-related effects, the perioperative inflammatory environment itself appears to directly influence Treg stability and phenotype (Wu D. et al., 2025). Elevated levels of inflammatory mediators following surgery may disrupt FoxP3 expression or interfere with Treg suppressive signaling, promoting a state of “functional fragility”(Handke et al., 2021). This concept is supported by experimental observations that perioperative inflammation compromises Treg-mediated immune tolerance, leading to sustained activation of innate immune cells and prolonged release of pro-inflammatory cytokines (Sokhi et al., 2020; Cross et al., 2021).

Importantly, alterations in Treg dynamics have been mechanistically linked to postoperative neuroinflammation and cognitive dysfunction (Zhou et al., 2023). In aged mice, postoperative Treg dysfunction correlated with increased blood–brain barrier permeability, enhanced leukocyte infiltration into the hippocampus, and exaggerated microglial activation. These central immune changes were accompanied by deficits in hippocampus-dependent learning and memory tasks, suggesting a causal relationship between peripheral Treg dysregulation and central cognitive outcomes (Meng X. et al., 2025).

Interventional studies further support the functional relevance of perioperative Treg alterations. Adoptive transfer of functional Tregs derived from young mice into aged surgical recipients partially restored blood–brain barrier integrity, reduced hippocampal inflammatory signaling, and improved postoperative cognitive performance. Conversely, experimental disruption of Treg signaling exacerbated neuroinflammatory responses, underscoring the protective role of intact Treg function during the perioperative period (Zhang et al., 2021; Beckmann et al., 2022).

**Figure 2:**
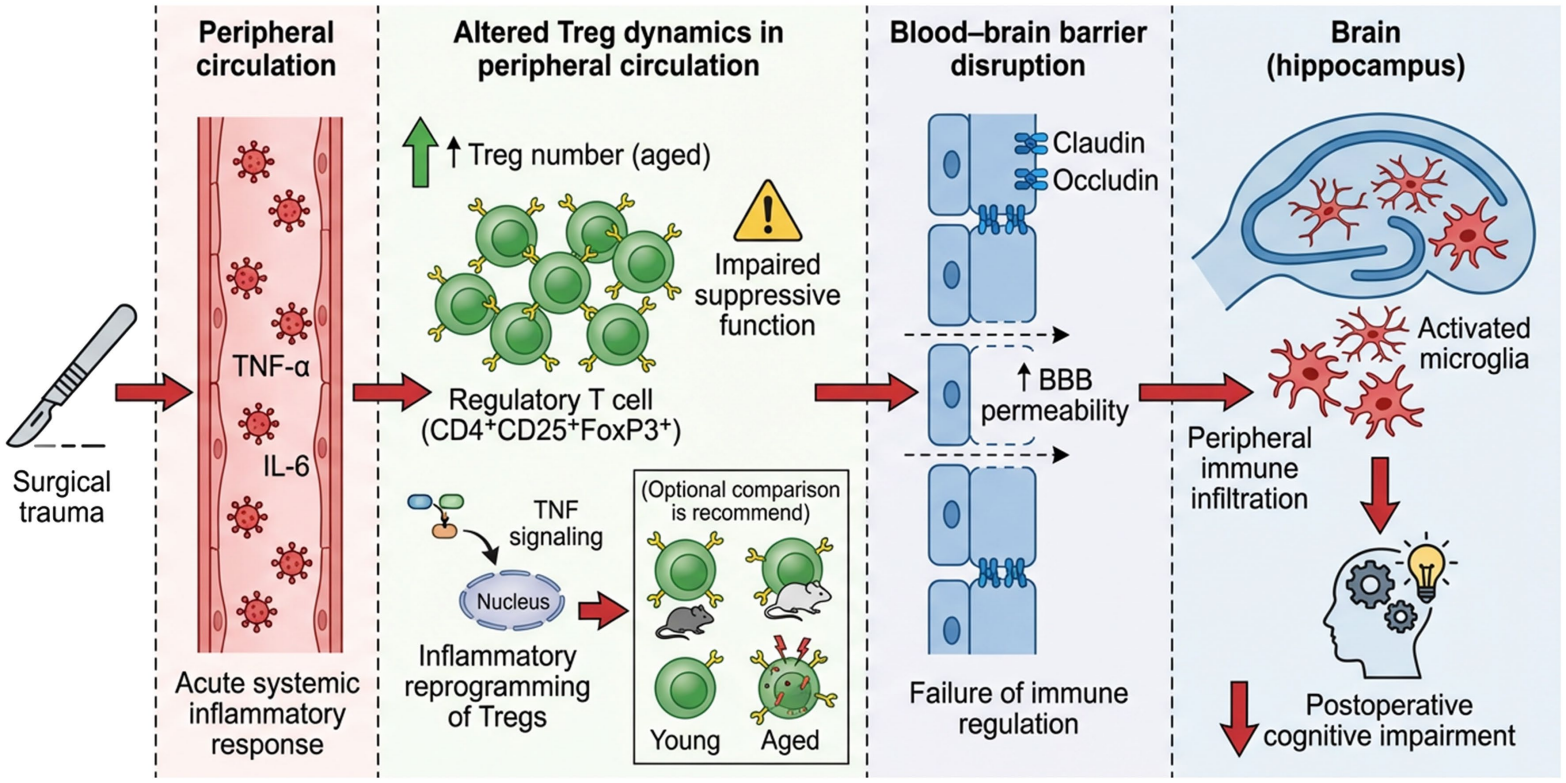
Perioperative dynamics of regulatory T cells following surgical trauma.

Collectively, these findings demonstrate that surgery induces complex alterations in Treg dynamics that extend beyond simple changes in cell number. Perioperative Treg dysfunction—particularly in aged subjects—represents a critical link between systemic inflammation and postoperative neurocognitive impairment. Understanding the mechanisms governing Treg stability and function after surgery may therefore provide important insights into age-related vulnerability to perioperative neurocognitive disorders and identify novel immunomodulatory targets for intervention (Figure 2).

Surgery induces an acute systemic inflammatory response characterized by elevated pro-inflammatory cytokines, such as tumor necrosis factor-*α* (TNF-α) and interleukin-6 (IL-6). In aged subjects, postoperative expansion of circulating CD4^+^CD25^+^FoxP3^+^ Tregs is frequently accompanied by impaired suppressive function, reflecting age-dependent functional fragility of Tregs. Dysfunctional perioperative Tregs fail to adequately restrain systemic inflammation, contributing to increased blood–brain barrier permeability, enhanced neuroimmune signaling, and vulnerability to postoperative cognitive impairment.

#### 3.2.2 Mechanistic insights into Treg-mediated modulation of perioperative neuroinflammation

Emerging preclinical evidence indicates that regulatory T cells modulate perioperative neurocognitive outcomes through multiple, interconnected mechanisms that operate across peripheral immune compartments and the central nervous system. Rather than acting through a single pathway, Tregs influence perioperative neuroinflammation via coordinated regulation of inflammatory signaling, blood–brain barrier integrity, and microglial activation states.

Beyond the well-recognized generic anti-inflammatory roles of Treg-derived IL-10 and TGF-*β*, several PND-specific molecular events have emerged from the included primary studies and merit explicit emphasis. First, the transcriptomic analysis of Tregs sorted from aged surgical mice by Zhou et al. (2023) revealed that the most strongly enriched pathways in postoperative Tregs were TNF signaling and cytokine–cytokine receptor interactions. This pattern indicates that perioperative Tregs do not simply lose suppressive output but undergo an active inflammatory reprogramming, consistent with the concept of “fragile” or “inflammatory” Tregs described in sepsis and trauma (Wu D. et al., 2025). Second, FoxP3 stability after surgery appears critically dependent on the metabolic environment: surgery-induced increases in lactate, oxidized LDL, and reactive oxygen species can destabilize FoxP3 via altered glycolysis–OXPHOS balance, suppression of TET2-mediated demethylation of the FOXP3 Treg-specific demethylated region (TSDR), and acetylation/ubiquitination switching of the FoxP3 protein (Zong et al., 2024; De la Torre Medina et al., 2025). Third, PND-specific chemokine signals—particularly the CCL2/CCR2 and CXCL10/CXCR3 axes—govern the differential trafficking of effector versus regulatory T cells across a perioperatively destabilized blood–brain barrier (Xian et al., 2021; Hermans et al., 2022; Peng et al., 2024), biasing the brain-resident T-cell compartment toward a proinflammatory phenotype when peripheral Treg function fails. These mechanistic threads are study-specific rather than textbook generalities and provide a more granular biological basis for the proposed Treg–PND link.

One of the primary mechanisms by which Tregs exert immunomodulatory effects is through the secretion of anti-inflammatory cytokines, particularly interleukin-10 (IL-10) and transforming growth factor-β (TGF-β) (Goldmann et al., 2024; Zong et al., 2024). In the perioperative context, these cytokines play a critical role in restraining excessive systemic inflammation induced by surgical trauma (Liu F. et al., 2022). Experimental models have demonstrated that impaired Treg function is associated with reduced anti-inflammatory cytokine signaling and a concomitant increase in pro-inflammatory mediators such as tumor necrosis factor-*α* (TNF-α) and interleukin-6 (IL-6) (Zong et al., 2024; Anandan and Narayanan, 2025). This imbalance favors a sustained inflammatory milieu that can propagate neuroinflammatory cascades.

Beyond cytokine-mediated effects, Tregs play a pivotal role in preserving blood–brain barrier (BBB) integrity following surgery. The BBB serves as a critical interface between peripheral immune responses and the central nervous system (Li et al., 2025; Qin et al., 2025). Experimental depletion or dysfunction of Tregs has been shown to exacerbate BBB permeability, leading to increased translocation of peripheral immune cells and inflammatory mediators into the brain parenchyma (Gao et al., 2022; Zhu et al., 2023). In aged mice subjected to surgical trauma, Treg dysfunction correlated with disruption of endothelial tight junction proteins and enhanced hippocampal exposure to circulating cytokines. Restoration of functional Tregs, either through adoptive transfer or immunomodulatory intervention, partially normalized BBB structure, highlighting a mechanistic link between Treg-mediated immune regulation and barrier integrity (Caplan et al., 2021; Gao et al., 2022).

Within the central nervous system, Tregs indirectly influence neuroinflammation by modulating microglial activation states (Ma et al., 2021; Park et al., 2022). Microglia represent the primary innate immune cells of the brain and are highly responsive to peripheral inflammatory signals. In perioperative models, exaggerated systemic inflammation and BBB disruption promote microglial activation toward a pro-inflammatory phenotype, characterized by increased production of neurotoxic mediators (Wang et al., 2026). Treg-mediated suppression of peripheral inflammation limits these signals, thereby attenuating microglial activation (Machhi et al., 2021). Moreover, anti-inflammatory cytokines associated with Treg activity have been implicated in promoting a shift toward an anti-inflammatory, neuroprotective microglial phenotype, which is more supportive of synaptic function and cognitive resilience (MaruYama et al., 2021; Ferreira et al., 2022).

Importantly, these mechanistic pathways converge to influence hippocampal structure and function, a brain region critical for learning and memory (Bayrak et al., 2022; Sammons et al., 2024). Excessive neuroinflammation resulting from Treg dysfunction has been associated with synaptic impairment, neuronal stress, and deficits in hippocampus-dependent cognitive tasks (He et al., 2021; Saadat et al., 2024). Conversely, experimental strategies that preserve or restore Treg function mitigate hippocampal inflammation and improve postoperative cognitive performance, providing functional validation of the mechanistic links between Tregs and perioperative neurocognitive outcomes (Uddin et al., 2022; Liu et al., 2025).

**Figure 3:**
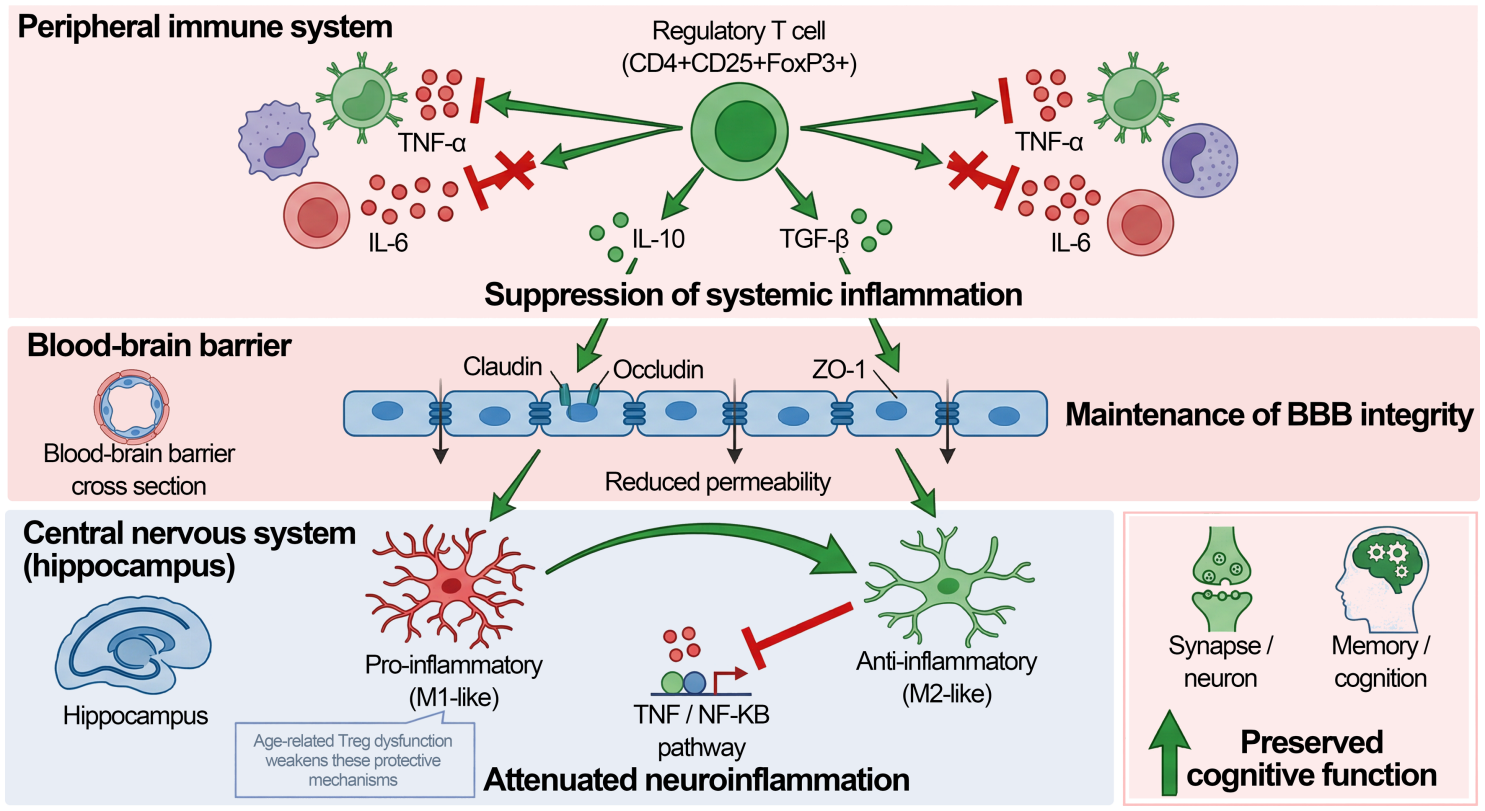
Regulatory T cell-mediated modulation of perioperative neuroinflammation and cognition.

Taken together, current evidence supports a multifaceted model in which regulatory T cells act as central regulators of perioperative immune–brain crosstalk. Through coordinated suppression of systemic inflammation, maintenance of blood–brain barrier integrity, and indirect modulation of microglial activation, Tregs play a crucial role in limiting surgery-induced neuroinflammation and protecting cognitive function (Wang et al., 2023; Xue et al., 2024). Disruption of these regulatory mechanisms—particularly in aged individuals—may therefore represent a key driver of vulnerability to perioperative neurocognitive disorders (Figure 3).

Tregs suppress excessive systemic inflammation by secreting anti-inflammatory cytokines, including interleukin-10 (IL-10) and transforming growth factor-β (TGF-β), thereby limiting the production of pro-inflammatory mediators. Treg-mediated immune regulation contributes to the preservation of blood–brain barrier integrity by maintaining endothelial tight junctions and restricting leukocyte infiltration into the central nervous system. Within the brain, reduced peripheral inflammatory signaling attenuates microglial activation and inhibits pro-inflammatory pathways, such as TNF/NF-κB signaling, ultimately resulting in reduced hippocampal neuroinflammation and preservation of cognitive function.

#### 3.2.3 Therapeutic modulation of Tregs in animal models

Among the included preclinical studies, Zhou et al. (2023) provided direct experimental evidence that therapeutic manipulation of Tregs can influence cognitive outcomes through flow cytometry and RNA sequencing of CD4 + CD25 + FoxP3 + cells (Table 3). Adoptive transfer of young functional Tregs into aged surgical recipients partially restored BBB structure and improved postoperative cognition, highlighting a direct causal link between Treg function and perioperative neurocognitive outcomes. Three additional studies (Cibelli et al., 2010; Terrando et al., 2011; Vacas et al., 2014) offer contextual mechanistic evidence supporting the broader neuroimmune framework within which Treg dysfunction operates, although these did not directly quantify Treg frequency or function (Table 3, denoted †). For example, adoptive transfer of young functional Tregs into aged animals partially restored BBB structure and improved postoperative cognition, highlighting a potential immunomodulatory intervention route (Zhou et al., 2023).

### 3.3 Clinical evidence linking Tregs to PND

Six clinical studies investigating the association between regulatory T cells and perioperative neurocognitive disorders are summarized in Table 4. Of these, five directly assessed Treg frequency, phenotype, or function; one (Hshieh et al., 2018) was reclassified as contextual evidence during the inclusion audit (see Section 2.3). These studies encompass diverse surgical populations, perioperative time points, and immune assessment strategies, including measurements of regulatory T cell frequency, FoxP3 expression, and Th17/Treg balance. Although heterogeneous in design, the collective clinical evidence reveals recurring patterns of perioperative immune dysregulation that are associated with adverse cognitive outcomes, providing the foundation for the analyses presented below.

#### 3.3.1 Treg balance and postoperative cognitive outcomes

Clinical evidence increasingly suggests that perioperative imbalance between regulatory T cells and pro-inflammatory T cell subsets is closely associated with postoperative cognitive outcomes (Tian et al., 2015; Ying and Lin, 2025). Rather than absolute changes in regulatory T cell numbers alone, the relative balance between immunoregulatory and pro-inflammatory responses—often reflected by the Th17/Treg ratio—appears to be a more robust indicator of perioperative immune homeostasis and cognitive vulnerability (Hanidziar and Koulmanda, 2010; Ashoor and Najafian, 2012).

Prospective and observational clinical studies have consistently reported that patients who develop postoperative cognitive dysfunction or delirium exhibit exaggerated perioperative inflammatory responses accompanied by dysregulated adaptive immunity (Needham et al., 2022; Ying and Lin, 2025). In these patients, reduced regulatory T cell activity, decreased FoxP3 expression, or an increased Th17/Treg ratio has been observed during the early postoperative period. Such immune alterations are frequently paralleled by elevated circulating pro-inflammatory cytokines, including interleukin-6 and tumor necrosis factor-α, which have been independently linked to postoperative neurocognitive impairment (Morishita et al., 2026; Yang et al., 2026).

Mechanistically, an increased Th17/Treg ratio reflects a shift toward a pro-inflammatory immune phenotype that may amplify systemic inflammatory signaling following surgery (Gowdy et al., 2012). Th17 cells are potent producers of interleukin-17, a cytokine known to promote endothelial activation and compromise vascular barrier integrity (Huang et al., 2021; Carvajal Gonczi et al., 2023). In the perioperative context, excessive Th17-mediated inflammation may facilitate blood–brain barrier disruption, thereby enhancing the penetration of peripheral inflammatory mediators into the central nervous system (Hermans et al., 2022; Peng et al., 2024). In contrast, regulatory T cells counterbalance these effects through anti-inflammatory cytokine production and immune suppression, suggesting that loss of this regulatory control may predispose patients to postoperative neuroinflammation (Dikiy and Rudensky, 2023).

To facilitate quantitative comparison across the clinical studies, the absolute Th17 frequencies, Treg frequencies, and Th17/Treg ratio values reported in each study—together with the assay platform and timing of measurement—are summarized in Table 5.

**Table 5:** Th17/Treg ratio summary across clinical studies.

Study (year)	Assay platform	Sampling timepoint(s)	Th17 frequency (% of CD4^+^)	Treg frequency (% of CD4^+^)	Th17/Treg ratio (POCD direction)
Zhao et al. (2023b)	Flow cytometry (protocol)	Pre-op, POD1, POD3, POD7	Protocol—assays pre-specified	Protocol—assays pre-specified	Pre-specified secondary outcome
Zhang et al. (2021)	Flow cytometry, FoxP3^+^	Pre-op, POD1, POD3, POD7	Not reported	↓ FoxP3^+^ in POCD	Inferred ↑ (qualitative)
Wang J. et al. (2022)	Flow cytometry; IHC; ELISA	Pre-op, POD1, POD3	↑ In POCD	↓ In POCD	↑ In POCD (*p* < 0.05)
Verdonk et al. (2024)	Mass cytometry (CyTOF), single-cell	Pre-op, POD1, POD7	Trend ↑ in POCD	↓ Functional capacity in POCD	Not directly reported; immune signature derived

Clinical interventional studies provide indirect support for a causal relationship between Treg balance and postoperative cognitive outcomes. In a randomized controlled trial involving elderly patients undergoing orthopedic surgery, Zhao et al. demonstrated that perioperative administration of dexmedetomidine significantly reduced the incidence of postoperative cognitive dysfunction (Zhao et al., 2023b). This neuroprotective effect was accompanied by partial normalization of the Th17/Treg balance; however, dexmedetomidine’s pleiotropic actions—including α2-adrenergic sympatholysis, opioid-sparing, sedation-mediated stress reduction, and microglial α2A-receptor signaling—preclude attribution of the cognitive benefit solely to Treg modulation. The Th17/Treg shift observed in this trial should therefore be interpreted as one of several plausible mediating pathways rather than as confirmation of a Treg-mediated mechanism (Wang D. et al., 2025; Zhang X. et al., 2025; Zhao L. et al., 2025).

**Figure 4:**
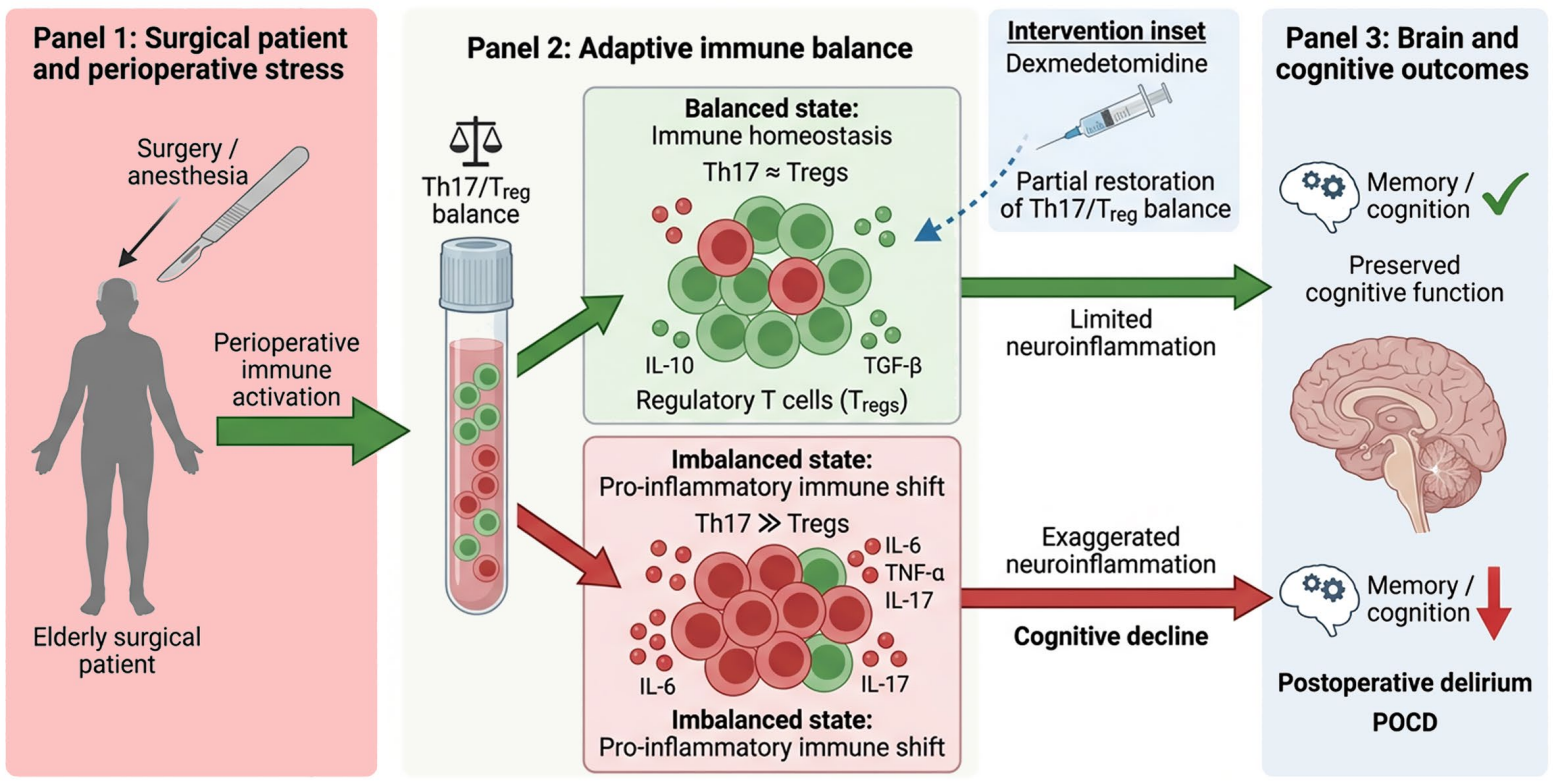
Clinical evidence for regulatory T cell involvement in perioperative neurocognitive disorders.

Additional observational studies further indicate that perioperative reductions in regulatory T cell markers, such as FoxP3 (Zhao et al., 2023a) expression, are associated with worse cognitive outcomes. Patients with postoperative delirium or cognitive decline often exhibit immune profiles characterized by heightened inflammatory signaling and diminished regulatory capacity (Saxena et al., 2021a; Lu et al., 2022). While these studies do not establish causality, they reinforce the concept that impaired immunoregulatory balance may contribute to sustained postoperative inflammation and neurocognitive vulnerability (Figure 4).

In elderly surgical patients, perioperative immune profiling reveals that an imbalance between pro-inflammatory T helper 17 (Th17) cells and regulatory T cells—often reflected by an increased Th17/Treg ratio—is associated with heightened systemic inflammation and increased risk of postoperative delirium and postoperative cognitive dysfunction. Clinical interventional studies indicate that immunomodulatory strategies, such as perioperative dexmedetomidine administration, may partially restore Th17/Treg balance and are associated with improved postoperative cognitive outcomes.

Notably, patient-related factors such as advanced age and comorbidities may exacerbate perioperative immune dysregulation (Yan et al., 2025). Immunosenescence is associated with reduced Treg functional plasticity and a predisposition toward pro-inflammatory immune responses, potentially amplifying the impact of surgical stress on immune balance (Lathe and St Clair, 2023; Doherty et al., 2025; Maity et al., 2026). This age-dependent vulnerability may partially explain the higher incidence of postoperative neurocognitive disorders observed in elderly populations (Tang et al., 2022; Feng et al., 2024; Saxena et al., 2025).

Collectively, current clinical evidence supports an association between perioperative Treg imbalance and adverse postoperative cognitive outcomes. While direct mechanistic studies in humans remain limited, converging data suggest that disruption of regulatory immune balance facilitates systemic inflammation and neuroinflammatory signaling, thereby increasing the risk of postoperative cognitive dysfunction (Kong et al., 2024b; Zhang L. et al., 2025; Zou et al., 2025). Future clinical studies incorporating longitudinal immune profiling and standardized cognitive assessments will be essential to clarify causality and to determine whether targeted modulation of Treg balance can serve as an effective strategy for preventing perioperative neurocognitive disorders (Jia et al., 2023; Koh and Joo, 2025; Wang J. L. et al., 2025).

Importantly, despite the consistent directional association between an elevated Th17/Treg ratio and adverse postoperative cognitive outcomes, the Th17/Treg ratio cannot at present be recommended as an independent predictive or diagnostic biomarker for PND. Three limitations preclude this: (i) population reference ranges have not been established; (ii) measurement methods (flow cytometric gating strategies, surface vs. intracellular FoxP3, frequency vs. absolute count) and sampling timepoints vary substantially across studies, undermining cross-study comparability; and (iii) the available studies are individually underpowered for biomarker validation. Adequately powered, methodologically harmonized prospective cohorts are needed before clinical biomarker translation can be considered.

#### 3.3.2 Immune cell subset studies in POD

While not all clinical work explicitly focused on Tregs, comprehensive profiling of immune cell subsets in patients with postoperative delirium after cardiac surgery (Elias et al., 2024) found altered innate and adaptive immune activation patterns, supporting the concept that immune dysfunction contributes to postoperative cognitive complications. During revision we re-audited the clinical citations against our pre-specified inclusion criterion that the study directly assessed Treg frequency, phenotype, or function in the perioperative period. The Hshieh et al. (2018) entry, which evaluates the multicomponent Hospital Elder Life Programme (HELP) for delirium prevention rather than perioperative Treg profiling, has been reclassified as contextual evidence and is no longer counted within the primary Treg-profiling synthesis pool. Its inclusion in the narrative serves only to illustrate the broader recognition of immune–cognitive interactions in postoperative delirium.

### 3.4 Risk of bias assessment

Risk of bias assessment was performed by two reviewers independently using the SYRCLE risk-of-bias tool for animal studies and the Newcastle–Ottawa Scale (NOS) for clinical observational studies, with disagreements resolved by consensus.

Among the four included animal studies (Table 1), risk of bias was generally judged “unclear” for the items related to sequence generation, allocation concealment, and blinding of caregivers and outcome assessors, reflecting incomplete reporting rather than evidence of methodological flaws. The domains “random housing,” “incomplete outcome data,” and “selective reporting” were generally judged at low risk. The most robust study with respect to mechanistic and behavioral endpoints was Zhou et al. (2023), which reported both flow-cytometric and transcriptomic data with explicit randomization and blinding.

Among the six clinical studies (Table 2), Newcastle–Ottawa Scale scores ranged from 5 to 8 out of a maximum of 9. Most studies adequately defined the surgical cohort and ascertained postoperative cognitive outcomes through standardized neuropsychological batteries; however, comparability scores varied because of inconsistent adjustment for age, baseline cognitive status, and surgical risk. Outcome ascertainment was generally adequate, with one study scored lower for unclear timing of cognitive assessment.

Overall, the certainty of evidence linking perioperative Treg dysregulation to PND was judged as low-to-moderate. The directional consistency of findings across heterogeneous animal models and clinical cohorts strengthens biological plausibility, but the small evidence base, predominance of observational designs, and methodological heterogeneity preclude high-certainty causal inference. These risk-of-bias considerations are integrated into the interpretive framework in the Discussion.

## 4 Discussion

### 4.1 Principal findings

This systematic review integrates preclinical and clinical evidence implicating regulatory T cells as critical modulators of perioperative neurocognitive disorders. Rather than acting as isolated immune effectors, Tregs emerge as potential determinants of whether the inflammatory response to surgical trauma is effectively constrained or evolves into sustained neuroinflammatory pathology (Caplan et al., 2021; Horner et al., 2023). This synthesis suggests that perioperative cognitive vulnerability may reflect not simply the magnitude of inflammation, but the failure of immunoregulatory mechanisms to restore immune homeostasis.

Integrated conceptual model illustrating the role of regulatory T cells in the pathophysiology of perioperative neurocognitive disorders. Surgical stress induces systemic inflammation and disrupts immune homeostasis, leading to Treg dysfunction or imbalance. Impaired Treg-mediated immune regulation promotes blood–brain barrier disruption, microglial activation, and hippocampal neuroinflammation, ultimately resulting in cognitive impairment. Potential therapeutic intervention points include enhancement of Treg function, targeted anti-inflammatory therapies, and perioperative immune modulation strategies aimed at preserving neuroimmune homeostasis.

**Figure 5:**
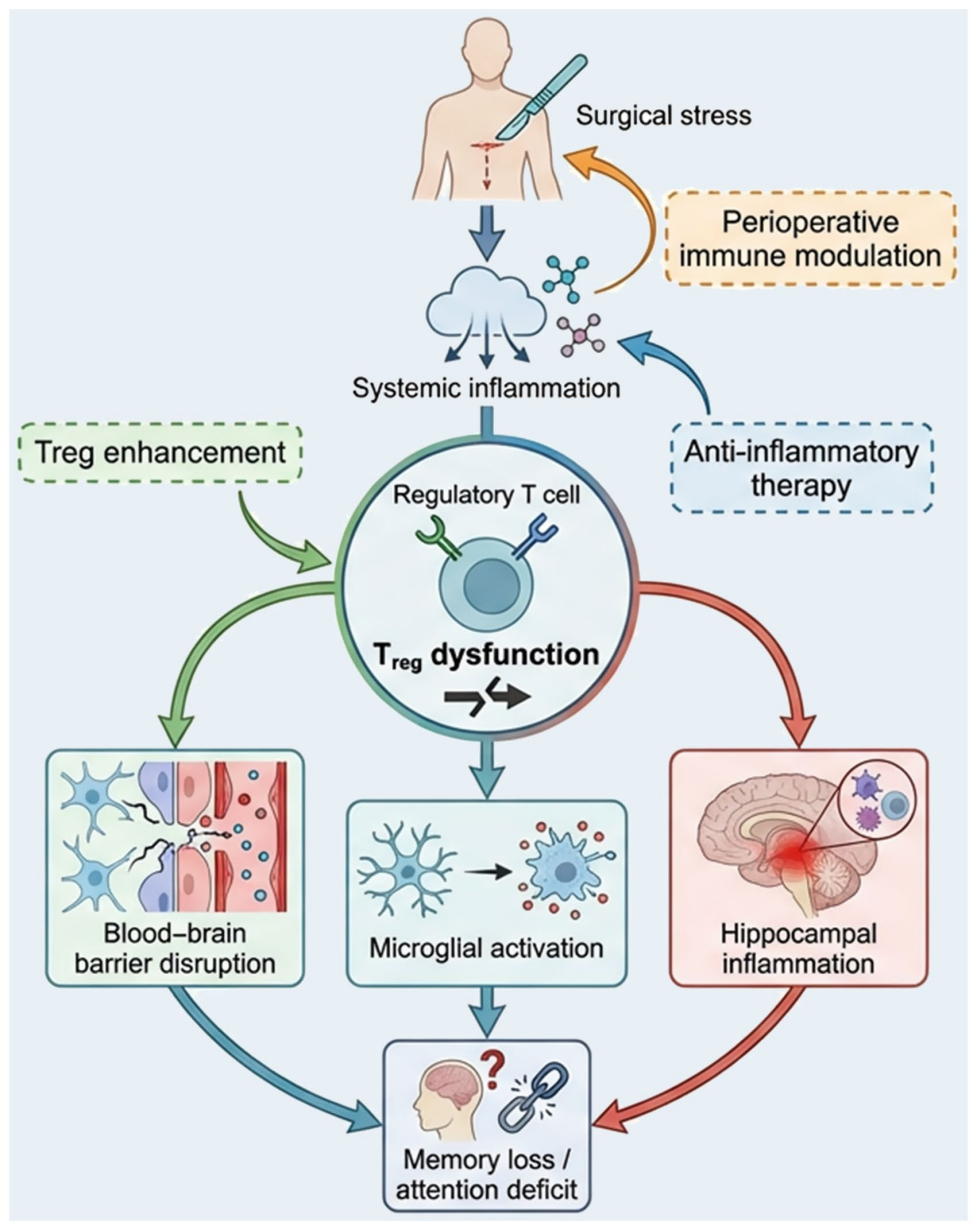
Regulatory T cells in PND: pathophysiology and therapeutic targets.

Preclinical studies, particularly in aged animals, consistently demonstrate functional impairment of Tregs in association with heightened inflammatory gene signatures, blood–brain barrier disruption, and adverse cognitive outcomes (Figure 5). These findings support the concept that age-related immunoregulatory decline may lower the threshold for postoperative neuroinflammation (Kong et al., 2024a). Importantly, experimental restoration of Treg function partially reverses these effects, indicating a contributory—but not exclusive—role for Tregs in perioperative cognitive resilience. At the same time, these models highlight an unresolved paradox: increases in Treg number do not necessarily equate to preserved suppressive capacity, underscoring the importance of functional, rather than numerical, immune regulation (Xian et al., 2021; Bai et al., 2022; John et al., 2022).

Clinical evidence, although limited and heterogeneous, converges on a similar pattern of dysregulated adaptive immunity in patients who develop postoperative cognitive dysfunction or delirium (Liu L. et al., 2022; Li et al., 2023, 2024). Elevated Th17/Treg ratios and reduced FoxP3 expression have been repeatedly observed in association with adverse cognitive outcomes (Wang et al., 2023; Qu et al., 2024; Zhu et al., 2025). However, these associations remain largely correlative, and it remains unclear whether Treg dysregulation is a driver of postoperative neurocognitive decline or a marker of broader immune dysfunction (Ying and Lin, 2025). Interventional observations, such as the association between dexmedetomidine administration and partial normalization of regulatory immune balance, should therefore be interpreted as mechanistic clues rather than definitive evidence of causality, given the pleiotropic actions of such agents.

An integrated mechanistic framework synthesizing these findings is presented in Table 6. Within this framework, surgical injury initiates systemic inflammatory signaling that perturbs immunoregulatory balance, favoring pro-inflammatory phenotypes and increasing endothelial and blood–brain barrier vulnerability (Villa et al., 2025). The ensuing bidirectional crosstalk between peripheral immune dysregulation and central neuroimmune activation may sustain cognitive impairment beyond the immediate postoperative period (Zeidan et al., 2026). Notably, this model does not imply a singular pathway to perioperative neurocognitive disorders but instead supports the existence of immunologically distinct trajectories, only some of which may be Treg-dependent.

**Table 6:** Summary of proposed mechanisms and therapeutic implications of regulatory T cells in perioperative neurocognitive disorders.

Pathophysiological process	Role of Tregs	Molecular mediators	Evidence source	Therapeutic implications
Systemic inflammation	Suppression of excessive immune activation	IL-10, TGF-β	Animal and clinical studies	Treg-enhancing immunomodulation
Blood–brain barrier disruption	Maintenance of endothelial integrity	Tight junction proteins	Animal models	Perioperative immune protection strategies
Microglial activation	Promotion of anti-inflammatory phenotype	TNF/NF-κB inhibition	Preclinical studies	Targeted anti-inflammatory therapies

### 4.2 Bridging the preclinical–clinical divide

A meaningful integration of preclinical and clinical evidence requires explicit recognition of three categories of discordance. First, Treg subsets are not biologically equivalent across species. Rodent studies overwhelmingly characterize CD4 + CD25 + FoxP3 + Tregs as a single population, whereas human Tregs encompass functionally distinct natural (thymic), induced (peripheral), naive, memory, tissue-resident, and effector subsets, including FOXP3 − regulatory phenotypes (Rubino et al., 2024). The human FOXP3 gene also produces multiple splice variants whose perioperative regulation has not been characterized. Inferences drawn from murine “Treg expansion with impaired suppression” therefore cannot be applied wholesale to humans without accounting for subset composition.

Second, the timing of immune assessment differs sharply. Rodent studies typically sample blood and CNS tissue at narrow postoperative windows of 6–72 h, capturing the acute neuroinflammatory response. Clinical studies, by contrast, generally restrict sampling to preoperative baselines and a small number of postoperative timepoints (days 1–7), missing both the early hyperacute phase and the longer resolution phase that may determine eventual cognitive trajectory (Hildenborg et al., 2022). Few clinical cohorts include both peripheral and CSF compartments, and none currently sample postoperative brain tissue—a fundamental asymmetry with animal models.

Third, cognitive outcome equivalence is a major source of conceptual leakage. “Postoperative cognitive testing” in rodents typically comprises the Morris water maze, fear conditioning, or Y-maze, which probe hippocampus-dependent spatial or contextual memory under stress (Sammons et al., 2024). Clinical PND criteria, by contrast, integrate DSM-based delirium diagnosis (with attention, awareness, and arousal components) and ISPOCD-style neuropsychological batteries assessing executive function, attention, memory, processing speed, and language (Mahanna-Gabrielli et al., 2019; Berger et al., 2023). These are not measurements of the same construct and cannot be interpreted as direct surrogates of each other.

Recognizing these discordances does not invalidate the proposed Treg framework; instead, it constrains the inferential weight of cross-species extrapolation and clarifies which preclinical claims (e.g., adoptive Treg transfer reverses BBB disruption) remain hypothesis-generating rather than confirmed in humans.

### 4.3 Dexmedetomidine and the limits of Treg-specific attribution

Dexmedetomidine is the most frequently cited pharmacological example linking perioperative Treg balance to cognitive outcomes (Zhao et al., 2023a; Zhao et al., 2023b). The agent’s observed normalization of the Th17/Treg ratio in elderly orthopedic patients is mechanistically plausible, but a Treg-specific interpretation is unjustified for at least four reasons. (i) Dexmedetomidine is a selective *α*2-adrenoceptor agonist with intrinsic anti-inflammatory effects that operate via sympatholysis and macrophage α2A signaling, both independent of adaptive immunity (Wang J. et al., 2022). (ii) Dexmedetomidine is opioid-sparing; reduced perioperative opioid exposure is itself associated with lower PND risk, and opioids independently modulate microglial activation (Muscat et al., 2023). (iii) Dexmedetomidine reduces sedation-associated neuroendocrine stress, attenuating cortisol-mediated effects on cognition and immunity. (iv) Direct *in vitro* effects of dexmedetomidine on regulatory T cell metabolism, FoxP3 stability, or Th17 differentiation have been reported but not standardized (Zhang X. et al., 2025; Zhao L. et al., 2025). Causal mediation analysis or trials with pre-specified Treg readouts will be required to disentangle these competing mechanisms; until then, the cognitive benefit of dexmedetomidine should not be presented as evidence of a Treg-specific therapeutic axis.

### 4.4 Non-invasive brain stimulation as a physical immunomodulatory strategy in PND

Although our review focuses on Treg-targeted immunomodulation, regulatory immune balance is not the only modifiable lever in perioperative neurocognitive care. Non-invasive brain stimulation, and in particular repetitive transcranial magnetic stimulation (rTMS), has recently attracted attention as an emerging non-pharmacological strategy with potential immunomodulatory action in PND. rTMS uses pulsed magnetic fields to induce focal cortical excitability changes; over multiple sessions, these changes are accompanied by long-term potentiation–like plasticity and, importantly for the present discussion, by suppression of hippocampal and cortical proinflammatory cytokine release (TNF-α, IL-1β, IL-6) (Wu Z. et al., 2025).

Two recently published randomized controlled protocol papers provide an immediate translational frame. Wang J. et al. (2025) describe a single-blind RCT of perioperative rTMS over the right dorsolateral prefrontal cortex in elderly patients undergoing cardiac surgery, designed to assess whether stimulation reduces PND incidence. Wu Z. et al. (2025) describe a three-arm, sham-controlled RCT of 10 Hz rTMS over the left dorsolateral prefrontal cortex in elderly patients undergoing total knee arthroplasty, with co-primary outcomes of POCD incidence and changes in IL-1β, IL-6, TNF-α, and HMGB1. Although direct measurement of regulatory T cell endpoints is not part of either protocol, the inflammatory readouts overlap substantially with the proinflammatory milieu that drives Treg destabilization in perioperative settings.

A plausible biological rationale therefore exists for a Treg-relevant action of rTMS in PND: by suppressing perioperative microglial activation and IL-6/TNF-α release, cortical neuromodulation may indirectly preserve Treg suppressive competence and reduce the inflammatory pressure that promotes Treg “fragility.” However, this remains hypothesis-only. No published clinical or preclinical study to date has measured rTMS effects on Treg frequency, FoxP3 stability, or suppressive function in a perioperative context. Future trials should incorporate parallel Treg immunophenotyping into rTMS protocols for PND, to test whether the cognitive and inflammatory benefits of cortical neuromodulation are mediated, in part, through adaptive immune regulation.

### 4.5 Limitations and publication bias

Several limitations warrant emphasis. Much of the mechanistic evidence derives from animal models, and the extent to which Treg-centered pathways operate similarly in humans remains uncertain. Clinical studies vary widely in surgical context, timing of immune assessment, and cognitive outcome measures, limiting causal inference (Hildenborg et al., 2022). Moreover, perioperative neurocognitive disorders likely encompass biologically heterogeneous entities, and Treg dysfunction represents only one component of a complex network involving innate immunity, neurovascular integrity, and central glial responses (Arai et al., 2025).

The small evidence base (4 animal studies and 6 clinical studies) warrants special caution. A symmetric inclusion audit was applied to both preclinical and clinical pools: studies that did not directly assess Treg frequency, phenotype, or function were reclassified as contextual mechanistic evidence (1 animal study directly assessed Tregs; 5 clinical studies directly assessed Tregs). This transparent reclassification preserves the narrative contribution of contextual studies while clearly delineating the strength of direct evidence. We did not perform funnel-plot or Egger’s test analyses because no quantitative meta-analysis was undertaken. Nevertheless, we recognize that publication bias is plausible in this small pool: positive associations between immune dysregulation and PND may be over-represented relative to null findings, particularly in single-center observational cohorts. We searched ClinicalTrials.gov and the WHO ICTRP for completed but unpublished perioperative immune-cognition trials; no such studies that would change our conclusions were identified, but the existence of small unpublished studies cannot be excluded. Additional review-process limitations include the absence of formal GRADE certainty rating, and the constraint that only English-language full-text articles were assessed.

### 4.6 Translational roadmap

Translating the proposed Treg-centric framework into clinical benefit requires four sequential steps. First, standardized, longitudinal multiparameter Treg immunophenotyping (including FoxP3 stability markers, Helios, CTLA-4, CD45RA, and TSDR demethylation) should be embedded into ongoing perioperative biobanks at high-risk surgical centers. Second, human ex vivo functional studies of regulatory T cells isolated from elderly surgical patients with and without PND should test whether suppressive capacity, not just frequency, distinguishes vulnerable phenotypes (Faridar et al., 2022). Third, early-phase clinical studies of Treg-targeted strategies—low-dose IL-2, CD28 superagonists with appropriate safety guardrails, or autologous polyclonal Treg infusion—could be tested in high-risk surgical populations using PND incidence as a secondary outcome (Zhao Y. et al., 2025). Fourth, immune profiling should be integrated as a pre-specified mechanistic sub-study in pragmatic trials of established PND interventions (dexmedetomidine, EEG-guided anesthesia, rTMS, multimodal pre-habilitation) to delineate which mechanisms account for clinical effects (Wang J. et al., 2025; Wu Z. et al., 2025). Together, these steps would transform the current pattern of correlative observation into a coherent translational program.

These observations carry important translational implications. Perioperative immune profiling may enable identification of patient subgroups with impaired immunoregulatory capacity who are particularly susceptible to neurocognitive complications (Ferrante et al., 2024; Rubino et al., 2024). More fundamentally, they suggest that future clinical trials should move beyond inflammation-centric endpoints and incorporate regulatory immune metrics as stratification variables or mechanistic readouts (Garduno and Martín-Loeches, 2024). Such an approach may help reconcile inconsistent clinical trial outcomes and enable more targeted intervention strategies.

Looking forward, the field may benefit from reframing perioperative neurocognitive disorders as immunologically heterogeneous syndromes rather than a single entity. Longitudinal studies integrating standardized cognitive assessments with dynamic, high-resolution immune profiling will be essential to delineate causal pathways and identify immune endotypes of vulnerability. Within this framework, regulatory T cells may serve not as universal therapeutic targets, but as critical indicators of immune resilience that help determine individual susceptibility to postoperative neurocognitive decline.

## 5 Conclusion

This systematic review consolidates evidence that regulatory T cells are integral to the neuroimmune mechanisms underlying perioperative neurocognitive disorders. In both animal models and clinical settings, dysregulated Treg responses are associated with enhanced inflammation and cognitive impairment, whereas strategies that preserve or restore regulatory T cell function exhibit neuroprotective effects. These insights support further exploration of Treg-targeted immunomodulation as a promising approach to prevent or ameliorate PND.

## Data Availability

The datasets presented in this study can be found in online repositories. The names of the repository/repositories and accession number(s) can be found in the article/Supplementary material.
