# Supplementary material for: Regulatory T cells in perioperative neurocognitive disorders: a systematic review with structured narrative synthesis from molecular mechanisms to clinical translation
Source: Front Mol Neurosci. 2026 Jul 16;19:1856316. doi: 10.3389/fnmol.2026.1856316 (PMC13422467; doi:10.3389/fnmol.2026.1856316)

**S1.1 Protocol overview and PICO framework**

This systematic review with structured narrative synthesis was conducted in accordance with the Preferred Reporting Items for Systematic Reviews and Meta-Analyses (PRISMA) 2020 guidelines (Page et al., 2021, *BMJ* 372:n71, doi: 10.1136/bmj.n71). A predefined internal protocol was prepared prior to literature screening; the review was not registered in PROSPERO. The internal protocol document is available from the corresponding author on reasonable request.

The review question was framed using a modified PICO framework appropriate for a mechanistic synthesis spanning preclinical and clinical evidence:

| **PICO element** | **Definition for this review** |
| --- | --- |
| **Population** | Animal models undergoing surgical procedures OR adult human surgical patients (any surgical specialty), with or without age stratification. |
| **Intervention / Exposure** | Quantitative or qualitative assessment of regulatory T cells (Tregs) — including CD4^+^CD25^+^FoxP3^+^ Tregs and recognised subsets — with respect to frequency, phenotype, suppressive function, or experimental modulation in the perioperative period. Pharmacological or biological agents that explicitly target Treg balance were also eligible. |
| **Comparator** | Non-surgical controls, sham-operated animals, age-matched controls, or pre-/post-surgical within-subject comparisons. |
| **Outcome** | Postoperative cognitive performance (behavioural tests in animals; standardised cognitive batteries, delirium criteria, or PND/POCD diagnoses in humans) and/or neuroinflammatory endpoints relevant to perioperative neurocognitive disorders (e.g., hippocampal cytokines, microglial activation markers, BBB integrity). |
| **Study design** | Original experimental and clinical studies (RCTs, prospective and observational cohort studies, case-control studies, mechanistic animal studies). Excluded: reviews, editorials, commentaries, letters, protocols without data, conference abstracts. |
| **Time frame** | Studies in which immune and cognitive assessments were performed within the perioperative period (pre-, intra-, or postoperative, up to 12 months post-surgery). |

**S1.2 Database-specific search strategies**

Searches were performed across seven sources. All searches were executed on **17 May 2026**. No date or language restrictions were applied at the database level, though only English-language full-text articles were ultimately assessed at the eligibility stage.

**S1.2.1 PubMed / MEDLINE**

*Source: PubMed (NLM, MEDLINE) · Search field: All Fields with MeSH explosion · Date executed: 17 May 2026*

**#1**"T-Lymphocytes, Regulatory"[MeSH Terms] **#2**"Forkhead Transcription Factors"[MeSH Terms] **#3**("regulatory T cell*"[tiab] OR "Treg"[tiab] OR "Tregs"[tiab] OR "FoxP3"[tiab] OR "FOXP3"[tiab] OR "CD4+CD25+"[tiab]) **#4**#1 OR #2 OR #3 **#5**"Cognitive Dysfunction"[MeSH Terms] **#6**"Postoperative Cognitive Complications"[MeSH Terms] **#7**"Delirium"[MeSH Terms] **#8**("perioperative neurocognitive disorder*"[tiab] OR "PND"[tiab] OR "postoperative cognitive dysfunction"[tiab] OR "POCD"[tiab] OR "postoperative delirium"[tiab] OR "POD"[tiab] OR "postoperative cognitive decline"[tiab] OR "post-operative cognitive*"[tiab]) **#9**#5 OR #6 OR #7 OR #8 **#10**"Perioperative Period"[MeSH Terms] OR "Postoperative Period"[MeSH Terms] OR "Anesthesia"[MeSH Terms] OR "Surgical Procedures, Operative"[MeSH Terms] **#11**("perioperative"[tiab] OR "postoperative"[tiab] OR "post-operative"[tiab] OR "surgery"[tiab] OR "surgical"[tiab] OR "anaesthesia"[tiab] OR "anesthesia"[tiab]) **#12**#10 OR #11 **#13**#4 AND #9 AND #12 **#14**Filters: English (applied at eligibility stage, not search stage

**S1.2.2 Embase (via Elsevier)**

*Source: Embase · Search field: ti,ab,kw + Emtree explosion · Date executed: 17 May 2026*

**#1**'regulatory t lymphocyte'/exp **#2**'forkhead box p3 protein'/exp **#3**('regulatory t cell*':ti,ab,kw OR 'treg':ti,ab,kw OR 'tregs':ti,ab,kw OR 'foxp3':ti,ab,kw OR 'cd4+cd25+':ti,ab,kw) **#4**#1 OR #2 OR #3 **#5**'postoperative cognitive dysfunction'/exp **#6**'postoperative delirium'/exp **#7**('perioperative neurocognitive disorder*':ti,ab,kw OR 'pocd':ti,ab,kw OR 'postoperative cognitive*':ti,ab,kw OR 'postoperative delirium':ti,ab,kw OR 'pnd':ti,ab,kw) **#8**#5 OR #6 OR #7 **#9**'perioperative period'/exp OR 'postoperative period'/exp OR 'anesthesia'/exp OR 'surgery'/exp **#10**('perioperative':ti,ab,kw OR 'postoperative':ti,ab,kw OR 'surgical':ti,ab,kw OR 'anaesthesia':ti,ab,kw OR 'anesthesia':ti,ab,kw) **#11**#9 OR #10 **#12**#4 AND #8 AND #11

**S1.2.3 Web of Science Core Collection**

*Source: Web of Science Core Collection (Clarivate) · Field: TS = Topic (title, abstract, author keywords, Keywords Plus) · Date executed: 17 May 2026*

**#1**TS=("regulatory T cell*" OR "T-reg*" OR "Treg" OR "Tregs" OR "FoxP3" OR "FOXP3" OR "CD4+CD25+") **#2**TS=("perioperative neurocognitive disorder*" OR "postoperative cognitive dysfunction" OR "POCD" OR "postoperative delirium" OR "POD" OR "postoperative cognitive decline" OR "PND") **#3**TS=("perioperative" OR "postoperative" OR "surgery" OR "surgical" OR "anaesthesia" OR "anesthesia") **#4**#1 AND #2 AND #3 **#5**Document Type: Article OR Review limit applied at screening stage.

**S1.2.4 Scopus**

*Source: Scopus (Elsevier) · Field: TITLE-ABS-KEY · Date executed: 17 May 2026*

TITLE-ABS-KEY ( ("regulatory T cell*" OR "Treg" OR "Tregs" OR "FoxP3" OR "FOXP3" OR "CD4+CD25+") AND ("perioperative neurocognitive disorder*" OR "POCD" OR "postoperative cognitive*" OR "postoperative delirium" OR "POD" OR "PND") AND ("perioperative" OR "postoperative" OR "surgery" OR "anaesthesia" OR "anesthesia") ) AND (LIMIT-TO (DOCTYPE,"ar") OR LIMIT-TO (DOCTYPE,"re"))

**S1.2.5 Cochrane Library — CENTRAL**

*Source: Cochrane Central Register of Controlled Trials (CENTRAL) · Field: title/abstract/keyword · Date executed: 17 May 2026*

**#1**("regulatory T cell*" OR "Treg" OR "FoxP3"):ti,ab,kw **#2**("perioperative neurocognitive" OR "POCD" OR "postoperative delirium" OR "postoperative cognitive"):ti,ab,kw **#3**("perioperative" OR "postoperative" OR "surgery" OR "anaesthesia" OR "anesthesia"):ti,ab,kw **#4**#1 AND #2 AND #3 (Trials only)

**S1.2.6 ClinicalTrials.gov**

*Source: ClinicalTrials.gov · Field: Condition or disease + Other terms · Date executed: 17 May 2026*

Condition or disease: Postoperative Cognitive Dysfunction OR Postoperative Delirium OR Perioperative Neurocognitive Disorders Other terms: regulatory T cell OR Treg OR FoxP3 OR Th17 OR immune modulation OR neuroinflammation Status filter applied at screening: Completed, Active (not recruiting), Recruiting.

**S1.2.7 WHO International Clinical Trials Registry Platform (ICTRP)**

*Source: WHO ICTRP · Date executed: 17 May 2026*

Search line: (regulatory T cell OR Treg OR FoxP3) AND (postoperative cognitive OR perioperative neurocognitive OR delirium)

**S1.2.8 Hand-searching and citation tracking**

Reference lists of all eligible articles and key narrative reviews were manually screened for additional relevant studies. Forward citation tracking was performed in Web of Science for the five animal studies meeting inclusion criteria to identify subsequent mechanistic studies. No additional unique records meeting eligibility were identified through hand-searching during the revision update.

**S1.3 Data extraction form**

A piloted data-extraction form was developed in Microsoft Excel and applied independently by two reviewers. The form captured the following data items for each included study:

| **Domain** | **Variables extracted** |
| --- | --- |
| **Bibliographic** | Author, year, country, journal, DOI, PMID, funding source, conflict-of-interest declaration. |
| **Design** | Study type (RCT, cohort, case-control, animal experimental); single-/multi-centre; blinding; sample size; statistical power statement. |
| **Population (animal)** | Species, strain, sex, age in weeks, weight, housing conditions, randomisation method. |
| **Population (human)** | n enrolled / analysed, age (mean ± SD or median [IQR]), sex distribution, comorbidities, ASA grade, baseline cognitive status (MMSE/MoCA/screening tool). |
| **Surgical procedure** | Specialty (orthopaedic, cardiac, abdominal, etc.), anaesthesia type, duration, blood loss, intraoperative events. |
| **Treg assessment** | Compartment (peripheral blood, lymph node, spleen, brain), assay (flow cytometry, qPCR, immunohistochemistry, sequencing), markers used, timing of sampling, gating strategy. |
| **Cognitive assessment** | Tool used (Morris water maze, fear conditioning, CAM-ICU, MoCA, neuropsychological battery), timing relative to surgery, criteria for diagnosing POCD/POD/PND. |
| **Inflammatory readouts** | Cytokines, microglial markers, BBB integrity measures, histology. |
| **Intervention** | Pharmacological agent, dose, schedule, route; cell adoptive transfer parameters; control condition. |
| **Key results** | Direction of effect, summary statistics, between-group difference, P value, effect size when reported. |
| **Risk of bias** | SYRCLE (animal) / Newcastle–Ottawa (clinical) domain-level judgments. |

**S1.4 SYRCLE risk-of-bias rubric for animal studies**

The SYRCLE tool for animal studies (Hooijmans et al., 2014, *BMC Med Res Methodol* 14:43) was used as published. Each of the ten domains was scored independently by two reviewers as Low risk (L), Unclear risk (U), or High risk (H). Disagreements were resolved by discussion. The aggregated assessments for the five included animal studies are reported in Table 4 of the main manuscript.

**SYRCLE domains used for assessment**

| **Item** | **Domain** | **Description (judgment criterion)** |
| --- | --- | --- |
| 1 | Sequence generation | Was the allocation sequence adequately generated and applied? |
| 2 | Baseline characteristics | Were baseline characteristics comparable across groups? |
| 3 | Allocation concealment | Was allocation adequately concealed? |
| 4 | Random housing | Were animals randomly housed during the experiment? |
| 5 | Blinding (caregivers) | Were caregivers/investigators blinded to group allocation? |
| 6 | Random outcome assessment | Were animals selected at random for outcome assessment? |
| 7 | Blinding (outcome assessor) | Was the outcome assessor blinded? |
| 8 | Incomplete outcome data | Were incomplete outcome data adequately addressed? |
| 9 | Selective outcome reporting | Are reports of the study free of selective outcome reporting? |
| 10 | Other sources of bias | Was the study apparently free of other problems? |

**S1.5 Newcastle–Ottawa Scale rubric for clinical studies**

The Newcastle–Ottawa Scale (NOS) was applied as published (Wells et al., 2014, Ottawa Hospital Research Institute), with the cohort-study version used for prospective and observational studies and the case-control version used where appropriate. Each study could earn up to 9 stars (4 for Selection, 2 for Comparability, 3 for Outcome). Quality bands: Good (7–9 stars), Moderate (4–6 stars), Poor (<4 stars). Domain-level judgments are reported in Table 5 of the main manuscript.

**NOS domains used (cohort study version)**

| **Category** | **Item** | **Maximum stars** |
| --- | --- | --- |
| **Selection** | Representativeness of the exposed cohort | ★ |
|  | Selection of the non-exposed cohort | ★ |
|  | Ascertainment of exposure (Treg measurement method) | ★ |
|  | Demonstration that the outcome (PND) was not present at the start | ★ |
| **Comparability** | Adjustment for age (primary confounder) | ★ |
|  | Adjustment for surgical risk, baseline cognition, or other key confounders | ★ |
| **Outcome** | Assessment of outcome (independent blind assessment / record linkage) | ★ |
|  | Sufficient follow-up duration | ★ |
|  | Adequacy of follow-up of cohorts | ★ |

**S1.6 Reviewer training and consensus protocol**

Two reviewers (YZ, KC) independently performed all screening, full-text assessment, data extraction, and risk-of-bias scoring. Prior to initiating screening, both reviewers piloted the data-extraction form and risk-of-bias rubrics on a calibration sample of five articles outside the final eligible pool. Calibration agreement was assessed informally; no formal kappa statistic was computed due to the small calibration sample. Disagreements during the actual screening process were resolved through discussion; a third reviewer (XD) adjudicated unresolved cases (none required adjudication in the final dataset). Reasons for full-text exclusion were recorded in real time using the standardised codes listed in Supplementary File S3.

**S1.7 Software and reporting tools**

**Reference management:** Zotero 7.0 was used for de-duplication and citation management.

Data extraction: Microsoft Excel (Version 16, Microsoft 365).

PRISMA flow diagram: Generated using the PRISMA 2020 flow diagram template (Page et al., 2021, Systematic Reviews 10:39).

Figure preparation: Adobe Illustrator 2025 for figure schematic; final figures exported at 300 dpi.

Reporting checklist: PRISMA 2020 checklist completed and provided as Supplementary File S2.

**Supplementary File S2. PRISMA 2020 Checklist**

| **Section / Topic** | **Item #** | **Checklist item** | **Location where item is reported** |
| --- | --- | --- | --- |
| **TITLE** | | | |
| **Title** | 1 | Identify the report as a systematic review. | Title page (Manuscript p.1) |
| **ABSTRACT** | | | |
| **Abstract** | 2 | See the PRISMA 2020 for Abstracts checklist. | Abstract (Manuscript p.2–3): structured into Background, Objective, Methods, Results, and Conclusion. |
| **INTRODUCTION** | | | |
| **Rationale** | 3 | Describe the rationale for the review in the context of existing knowledge. | Introduction §1 (paragraphs 1–3), Manuscript pp.4–5. |
| **Objectives** | 4 | Provide an explicit statement of the objective(s) or question(s) the review addresses. | Introduction final paragraph (Manuscript p.5) and Abstract “Objective.” |
| **METHODS** | | | |
| **Eligibility criteria** | 5 | Specify the inclusion and exclusion criteria for the review and how studies were grouped for the syntheses. | Methods Section 2.3 (Manuscript pp.7–8); Supplementary File S1, Section S1.1. |
| **Information sources** | 6 | Specify all databases, registers, websites, organisations, reference lists and other sources searched or consulted to identify studies. Specify the date when each source was last searched or consulted. | Methods Section 2.2 (Manuscript p.6-7). Last searched 17 May 2026. Sources listed in S1.2. |
| **Search strategy** | 7 | Present the full search strategies for all databases, registers and websites, including any filters and limits used. | Methods Section 2.2; full search strings in Supplementary File S1, Section S1.2. |
| **Selection process** | 8 | Specify the methods used to decide whether a study met the inclusion criteria of the review, including how many reviewers screened each record and each report retrieved, whether they worked independently, and if applicable, details of automation tools used in the process. | Methods Section 2.4 (Manuscript p.8): two independent reviewers; consensus resolution. See S1.6. |
| **Data collection process** | 9 | Specify the methods used to collect data from reports, including how many reviewers collected data from each report, whether they worked independently, any processes for obtaining or confirming data from study investigators, and if applicable, details of automation tools used in the process. | Methods Section 2.5 (Manuscript p.9): piloted form, dual independent extraction. See S1.3. |
| **Data items** | 10a | List and define all outcomes for which data were sought. | Methods Section 2.5: primary outcomes listed; all reported timepoints sought. |
|  | 10b | List and define all other variables for which data were sought. Describe any assumptions made about any missing or unclear information. | Methods Section 2.5; variables listed in S1.3. No imputation. |
| **Study risk of bias assessment** | 11 | Specify the methods used to assess risk of bias in the included studies, including details of the tool(s) used, how many reviewers assessed each study and whether they worked independently, and if applicable, details of automation tools used in the process. | Methods Section 2.6: SYRCLE (animal); Newcastle–Ottawa (clinical). See S1.4 and S1.5. |
| **Effect measures** | 12 | Specify for each outcome the effect measure(s) used in the synthesis or presentation of results. | Methods Section 2.7: no quantitative pooling; individual study estimates in Tables 1, 2, and 6. |
| **Synthesis methods** | 13a | Describe the processes used to decide which studies were eligible for each synthesis. | Methods Section 2.7: stratified by study type, compartment, and time frame. |
|  | 13b | Describe any methods required to prepare the data for presentation or synthesis. | Methods Section 2.7: data extracted as reported; no conversions. |
|  | 13c | Describe any methods used to tabulate or visually display results of individual studies and syntheses. | Tables 1–3, Table 6; Figures 1–5. |
|  | 13d | Describe any methods used to synthesize results and provide a rationale for the choice(s). | Methods Section 2.7: structured narrative synthesis; rationale provided. |
|  | 13e | Describe any methods used to explore possible causes of heterogeneity among study results. | Discussion Section 4.2. |
|  | 13f | Describe any sensitivity analyses conducted to assess robustness of the synthesized results. | Not applicable; no quantitative synthesis. |
| **Reporting bias assessment** | 14 | Describe any methods used to assess risk of bias due to missing results in a synthesis (arising from reporting biases). | Methods Section 2.6 and Discussion Section 4.5. |
| **Certainty assessment** | 15 | Describe any methods used to assess certainty (or confidence) in the body of evidence for an outcome. | Methods Section 2.6 and Results Section 3.4: qualitative certainty rating. |
| **RESULTS** | | | |
| **Study selection** | 16a | Describe the results of the search and selection process, from the number of records identified in the search to the number of studies included in the review, ideally using a flow diagram. | Results Section 3.1 and Figure 1: 3. 12 records, 46 full text, 12 included. |
|  | 16b | Cite studies that might appear to meet the inclusion criteria, but which were excluded, and explain why they were excluded. | Supplementary File S3 (Supplementary Table S1) with PMID and DOI for each excluded study. |
| **Study characteristics** | 17 | Cite each included study and present its characteristics. | Tables 1 and 2; Supplementary Table S3. |
| **Risk of bias in studies** | 18 | Present assessments of risk of bias for each included study. | Results Section 3.4; Tables 4 (SYRCLE) and 5 (Newcastle–Ottawa). |
| **Results of individual studies** | 19 | For all outcomes, present, for each study, summary statistics for each group and an effect estimate and its precision. | Tables 1, 2, and 6. |
| **Results of syntheses** | 20a | For each synthesis, briefly summarise the characteristics and risk of bias among contributing studies. | Results Sections 3.2–3.4. |
|  | 20b | Present results of all statistical syntheses conducted. | Not applicable; no meta-analysis. |
|  | 20c | Present results of all investigations of possible causes of heterogeneity among study results. | Discussion Section 4.2. |
|  | 20d | Present results of all sensitivity analyses conducted to assess the robustness of the synthesized results. | Not applicable. |
| **Reporting biases** | 21 | Present assessments of risk of bias due to missing results (arising from reporting biases) for each synthesis assessed. | Discussion Section 4.5. |
| **Certainty of evidence** | 22 | Present assessments of certainty (or confidence) in the body of evidence for each outcome assessed. | Results Section 3.4; Discussion Section 4.5. |
| **DISCUSSION** | | | |
| **Discussion** | 23a | Provide a general interpretation of the results in the context of other evidence. | Discussion Sections 4.1–4.2. |
|  | 23b | Discuss any limitations of the evidence included in the review. | Discussion Section 4.5. |
|  | 23c | Discuss any limitations of the review processes used. | Discussion Section 4.5. |
|  | 23d | Discuss implications of the results for practice, policy, and future research. | Discussion Sections 4.4 and 4.6; Conclusion. |
| **OTHER INFORMATION** | | | |
| **Registration and protocol** | 24a | Provide registration information for the review, including register name and registration number, or state that the review was not registered. | Methods Section 2.1: not registered in PROSPERO. |
|  | 24b | Indicate where the review protocol can be accessed, or state that a protocol was not prepared. | Internal protocol available from corresponding author; relevant elements in Supplementary File S1. |
|  | 24c | Describe and explain any amendments to information provided at registration or in the protocol. | No amendments after screening began. |
| **Support** | 25 | Describe sources of financial or non-financial support for the review, and the role of the funders or sponsors in the review. | Funding statement (Manuscript p.36). |
| **Competing interests** | 26 | Declare any competing interests of review authors. | Conflict of Interest Statement (Manuscript p.36-37). |
| **Availability of data, code and other materials** | 27 | Report which of the following are publicly available and where they can be found. | Data Availability Statement (Manuscript p.36); Supplementary Files S1–S3. |

The PRISMA 2020 statement: an updated guideline for reporting systematic reviews. *BMJ* 2021;372:n71. [doi:10.1136/bmj.n71](https://doi.org/10.1136/bmj.n71) Reproduced under CC BY 4.0.

**Supplementary File S3 / Supplementary Table S1. Excluded studies**

The following table lists representative full-text articles that were retrieved during the literature search but excluded from the final synthesis, with PMID and DOI provided for each entry. Each excluded study is mapped to one of the five pre-specified exclusion categories. The 22 entries shown represent the verified subset of full-text exclusions; the complete annotated screening log (n=34 exclusions) is available from the corresponding author on request.

Reason categories used:

**R1** — No Treg assessment (study did not measure Treg frequency, phenotype, or function)

**R2** — No cognitive outcome (no behavioural test in animals; no validated cognitive battery in humans)

**R3** — Non-perioperative context (study did not include a surgical/anaesthetic insult)

**R4** — Review/editorial/letter/abstract (no primary data)

**R5** — Duplicate publication or secondary analysis of an already-included dataset

| **#** | **Author (Year)** | **Title / Journal** | **Exclusion reason** | **PMID / DOI** |
| --- | --- | --- | --- | --- |
| 1 | Hshieh TT, Yang T, Gartaganis SL, Yue J, Inouye SK (2018) | *Hospital Elder Life Program: Systematic Review and Meta-analysis of Effectiveness* Am J Geriatr Psychiatry 26(10):1015-1033 | **no Treg** | PMID:30076080  doi:10.1016/j.jagp.2018.06.007 |
| 2 | Inouye SK, Westendorp RG, Saczynski JS (2014) | *Delirium in elderly people* Lancet 383(9920):911-922 | **review/editorial** | PMID:23992774  doi:10.1016/S0140-6736(13)60688-1 |
| 3 | Subramaniyan S, Terrando N (2019) | *Neuroinflammation and Perioperative Neurocognitive Disorders* Anesth Analg 128(4):781-788 | **review/editorial** | PMID:30883423  doi:10.1213/ANE.0000000000004053 |
| 4 | Skvarc DR, Berk M, Byrne LK, Dean OM, Dodd S, Lewis M, et al. (2018) | *Post-Operative Cognitive Dysfunction: An exploration of the inflammatory hypothesis and novel therapies* Neurosci Biobehav Rev 84:116-133 | **review/editorial** | PMID:29180259  doi:10.1016/j.neubiorev.2017.11.011 |
| 5 | Vasunilashorn SM, Ngo LH, Jones RN, Inouye SK, Hall KT, Gallagher J, et al. (2019) | *The Association Between C-Reactive Protein and Postoperative Delirium Differs by Catechol-O-Methyltransferase Genotype* Am J Geriatr Psychiatry 27(1):1-8 | **no Treg** | PMID:30424994  doi:10.1016/j.jagp.2018.09.007 |
| 6 | Vasunilashorn SM, Dillon ST, Inouye SK, Ngo LH, Fong TG, Jones RN, et al. (2017) | *High C-Reactive Protein Predicts Delirium Incidence, Duration, and Feature Severity After Major Noncardiac Surgery* J Am Geriatr Soc 65(8):e109-e116 | **no Treg** | PMID:28555781  doi:10.1111/jgs.14913 |
| 7 | Dillon ST, Vasunilashorn SM, Ngo L, Otu HH, Inouye SK, Jones RN, et al. (2017) | *Higher C-Reactive Protein Levels Predict Postoperative Delirium in Older Patients Undergoing Major Elective Surgery: A Longitudinal Nested Case-Control Study* Biol Psychiatry 81(2):145-153 | **no Treg** | PMID:27160518  doi:10.1016/j.biopsych.2016.03.2098 |
| 8 | Westhoff D, Witlox J, Koenderman L, Kalisvaart KJ, de Jonghe JFM, van Stijn MFM, et al. (2013) | *Preoperative cerebrospinal fluid cytokine levels and the risk of postoperative delirium in elderly hip fracture patients* J Neuroinflammation 10:122 | **no Treg** | PMID:24093540  doi:10.1186/1742-2094-10-122 |
| 9 | Liu X, Yu Y, Zhu S (2018) | *Inflammatory markers in postoperative delirium (POD) and cognitive dysfunction (POCD): A meta-analysis of observational studies* PLoS One 13(4):e0195659 | **review/editorial** | PMID:29641605  doi:10.1371/journal.pone.0195659 |
| 10 | Mao M, Wang LY, Zhu LY, Wang F, Ding Y, Tong JH, et al. (2022) | *Higher serum PGE2 is a predicative biomarker for postoperative delirium following elective orthopedic surgery in elderly patients* BMC Geriatr 22(1):685 | **no Treg** | PMID:35986284  doi:10.1186/s12877-022-03367-y |
| 11 | Lu W, Jiang Z, Huang J, Bian J, Yu X (2021) | *Preoperative Serum Metabolites and Potential Biomarkers for Perioperative Cognitive Decline in Elderly Patients* Front Psychiatry 12:665097 | **no Treg** | PMID:34093273  doi:10.3389/fpsyt.2021.665097 |
| 12 | Wang CG, Qin YF, Wan X, Song LC, Li ZJ, Li H (2018) | *Incidence and risk factors of postoperative delirium in the elderly patients with hip fracture* J Orthop Surg Res 13(1):186 | **no Treg** | PMID:30053904  doi:10.1186/s13018-018-0897-8 |
| 13 | Wang Y, Zhao L, Zhang C, An Q, Guo Q, Geng J, et al. (2021) | *Identification of risk factors for postoperative delirium in elderly patients with hip fractures by a risk stratification index model: A retrospective study* Brain Behav 11(12):e2420 | **no Treg** | PMID:34813683  doi:10.1002/brb3.2420 |
| 14 | Niu Q, Cai B, Huang Z-c, Shi Y-y, Wang L-l (2012) | *Disturbed Th17/Treg balance in patients with rheumatoid arthritis* Rheumatol Int 32(9):2731-2736 | **non-perioperative** | PMID:34813683  doi:10.1002/brb3.2420 |
| 15 | Kikodze N, Pantsulaia I, Chikovani T (2016) | *The role of T regulatory and Th17 cells in the pathogenesis of rheumatoid arthritis (Review)* Georgian Med News (261):62-68 | **review/editorial** | PMID:28132045 |
| 16 | Liu C, Yang H, Shi W, Wang T, Ruan Q (2018) | *MicroRNA-mediated regulation of T helper type 17/regulatory T-cell balance in autoimmune disease* Immunology 155(4):427-434 | **non-perioperative** | PMID:30133700  doi:10.1111/imm.12994 |
| 17 | Yan JB, Luo MM, Chen ZY, He BH (2020) | *The Function and Role of the Th17/Treg Cell Balance in Inflammatory Bowel Disease* J Immunol Res 2020:8813558 | **non-perioperative** | PMID:33381606  doi:10.1155/2020/8813558 |
| 18 | Astry B, Venkatesha SH, Moudgil KD (2015) | *Involvement of the IL-23/IL-17 axis and the Th17/Treg balance in the pathogenesis and control of autoimmune arthritis* Cytokine 74(1):54-61 | **review/editorial** | PMID:25595306  doi:10.1016/j.cyto.2014.11.020 |
| 19 | Kosmaczewska A, Ciszak L, Swierkot J, Szteblich A, Kosciow K, Wiland P (2014) | *Patients with the most advanced rheumatoid arthritis remain with Th1 systemic defects after TNF inhibitors treatment despite clinical improvement* Rheumatol Int 34(2):243-252 | **non-perioperative** | PMID:24096929  doi:10.1007/s00296-013-2885-y |
| 20 | Hridi SU, Franssen A, Jiang HR, Bushell TJ (2019) | *Interleukin-16 inhibits sodium channel function and GluA1 phosphorylation via CD4- and CD9-independent mechanisms to reduce hippocampal neuronal excitability and synaptic activity* Mol Cell Neurosci 95:71-78 | **non-perioperative** | PMID:30664929  doi:10.1016/j.mcn.2019.01.002 |
| 21 | Berg KS, Stenseth R, Pleym H, Wahba A, Videm V (2015) | *Neopterin predicts cardiac dysfunction following cardiac surgery* Interact Cardiovasc Thorac Surg 21(5):598-603 | **no cognitive** | PMID:26265068  doi:10.1093/icvts/ivv219 |
| 22 | Osse RJ, Fekkes D, Tulen JH, Wierdsma AI, Bogers AJ, van der Mast RC, Hengeveld MW (2012) | *High preoperative plasma neopterin predicts delirium after cardiac surgery in older adults* J Am Geriatr Soc 60(4):661-668 | **no Treg** | PMID:22316274  doi:10.1111/j.1532-5415.2011.03885.x |

**Summary of exclusion reasons**

| **Reason category** | **Verified entries shown** | **% of shown** |
| --- | --- | --- |
| **No Treg assessment (study did not measure Treg frequency, phenotype, or function)** | 10 | 45.5% |
| **No cognitive outcome (no behavioural test in animals; no validated cognitive battery in humans)** | 1 | 4.5% |
| **Non-perioperative context (study did not include a surgical/anaesthetic insult)** | 5 | 22.7% |
| **Review/editorial/letter/abstract (no primary data)** | 6 | 27.3% |
| **Duplicate publication or secondary analysis of an already-included dataset** | 0 | — |
| **Total verified entries shown** | **22** | **100%** |
| **Total full-text exclusions in screening log** | **34** | **—** |

**Supplementary Table S2. Glossary of abbreviations and definitions**

The following abbreviations and operational definitions are used throughout the manuscript and supplementary materials.

| **Abbreviation / Term** | **Definition / Operational meaning** |
| --- | --- |
| **PND** | Perioperative neurocognitive disorders — umbrella term covering postoperative delirium and longer-lasting postoperative cognitive decline, in accordance with the international Nomenclature Consensus (Evered et al., 2018). |
| **POCD** | Postoperative cognitive dysfunction — legacy term; clinically defined by significant decline from preoperative baseline on a standardised neuropsychological battery, typically assessed at 1 week, 3 months, and 12 months post-surgery. |
| **POD** | Postoperative delirium — acute (within 7 days of surgery) disturbance of attention, awareness, and cognition, diagnosed by DSM-5 criteria or instruments such as CAM-ICU or 3D-CAM. |
| **Treg** | Regulatory T cell — for the purpose of this review, defined as a CD4^+^CD25^+^FoxP3^+^ T-cell population with documented or inferred suppressive function. Recognised subsets include natural (thymic) Tregs, induced (peripheral) Tregs, naive Tregs, memory Tregs, tissue-resident Tregs, and effector Tregs. |
| **FoxP3 / FOXP3** | Forkhead box P3 transcription factor; master regulator of regulatory T-cell identity and function. Italicised *FOXP3* denotes the gene; FoxP3 denotes the protein. |
| **TSDR** | Treg-specific demethylated region — a conserved non-coding sequence within the *FOXP3* locus whose demethylation is required for stable FoxP3 expression. |
| **Th17/Treg ratio** | Ratio of circulating CD4^+^IL-17A^+^ Th17 cells to CD4^+^CD25^+^FoxP3^+^ Tregs, measured by flow cytometry as a surrogate of pro-inflammatory vs. regulatory immune balance. |
| **BBB** | Blood–brain barrier — the specialised endothelial interface restricting passage of cells and macromolecules between systemic circulation and the central nervous system. |
| **SYRCLE** | SYstematic Review Centre for Laboratory animal Experimentation risk-of-bias tool, used for methodological appraisal of animal studies. |
| **NOS** | Newcastle–Ottawa Scale — standardised tool for risk-of-bias assessment of non-randomised observational clinical studies. |
| **PRISMA 2020** | Preferred Reporting Items for Systematic Reviews and Meta-Analyses, 2020 statement (Page et al., 2021). |
| **ISPOCD** | International Study of Postoperative Cognitive Dysfunction — established the standard neuropsychological battery and statistical criteria for POCD diagnosis (Moller et al., 1998). |
| **MMSE / MoCA** | Mini-Mental State Examination / Montreal Cognitive Assessment — common bedside cognitive screening instruments used to characterise baseline cognition in perioperative cohorts. |
| **CAM-ICU / 3D-CAM** | Confusion Assessment Method for the Intensive Care Unit / 3-Minute Diagnostic CAM — validated bedside tools for delirium diagnosis. |
| **DAMPs** | Damage-associated molecular patterns — endogenous molecules released after tissue injury that activate innate immune signalling (e.g., HMGB1). |
| **HMGB1** | High-mobility group box 1 protein — a prototypical DAMP implicated in perioperative inflammation and PND. |
| **rTMS** | Repetitive transcranial magnetic stimulation — non-invasive brain stimulation modality being investigated as an immunomodulatory adjunct in PND. |
| **DLPFC** | Dorsolateral prefrontal cortex — cortical target of most rTMS protocols for cognitive disorders. |

**Supplementary Table S3. Treg phenotypic markers used across included studies**

Heterogeneity in the markers used to define and assess regulatory T cells across the included primary studies is summarised below. Each entry now includes its PubMed ID and Digital Object Identifier for direct cross-referencing with the main manuscript reference list. Cross-study comparison of “Treg frequency” should be interpreted with the heterogeneity in mind.

| **Study** | **PMID / DOI** | **Compartment** | **Surface markers** | **Intracellular markers** | **Functional assays** | **Notes** |
| --- | --- | --- | --- | --- | --- | --- |
| Zhou et al. (2023) | [PMID:36973694](https://pubmed.ncbi.nlm.nih.gov/36973694/) [doi:10.1186/s12974-023-02760-7](https://doi.org/10.1186/s12974-023-02760-7) | Spleen, blood, hippocampus (mouse) | CD4, CD25, CD127^low^ | FoxP3 | Suppression assay; RNA-seq | Most comprehensive Treg profiling among included studies; transcriptomic signature reported. |
| Terrando et al. (2011) | [PMID:22190370](https://pubmed.ncbi.nlm.nih.gov/22190370/) [doi:10.1002/ana.22664](https://doi.org/10.1002/ana.22664) | Spleen, blood (mouse) | CD4, CD25 | FoxP3 | Cytokine panel | Treg quantification embedded within wider neuroinflammation analysis. |
| Cibelli et al. (2010) | [PMID:20818791](https://pubmed.ncbi.nlm.nih.gov/20818791/) [doi:10.1002/ana.22082](https://doi.org/10.1002/ana.22082) | Spleen, blood (mouse) | CD4, CD25 | – | – | Primarily focused on IL-1β; Treg analysis limited. |
| Vacas et al. (2014) | [PMID:24632847](https://pubmed.ncbi.nlm.nih.gov/24632847/) [doi:10.1097/ALN.0000000000000045](https://doi.org/10.1097/ALN.0000000000000045) | Spleen, hippocampus (mouse) | CD4, CD25 | FoxP3 | – | Treg as adjunctive readout; primary focus on HMGB1 / bone-marrow-derived macrophages. |
| Safavynia & Goldstein (2019) | [PMID:30705643](https://pubmed.ncbi.nlm.nih.gov/30705643/) [doi:10.3389/fpsyt.2018.00752](https://doi.org/10.3389/fpsyt.2018.00752) | Review with mechanistic re-analysis | Variable across cited studies | Variable | Variable | Mechanistic review-with-data; aggregates earlier datasets. |
| Zhao W. et al. (2023a) | [PMID:37193184](https://pubmed.ncbi.nlm.nih.gov/37193184/) | Peripheral blood (human) | CD4, CD25, CD127^low^ | FoxP3 (intracellular) | Cytokine ELISA | Pre-op, POD1, POD3 sampling; Th17/Treg ratio reported. |
| Zhao W. et al. (2023b) | [PMID:36707873](https://pubmed.ncbi.nlm.nih.gov/36707873/) [doi:10.1186/s13063-023-07110-9](https://doi.org/10.1186/s13063-023-07110-9) | Peripheral blood (human) | CD4, CD25 | FoxP3 (intracellular) | Cytokine ELISA | Protocol paper for elderly orthopaedic RCT of dexmedetomidine and POCD. |
| Wang J. et al. (2022a) | [PMID:35468581](https://pubmed.ncbi.nlm.nih.gov/35468581/) [doi:10.1016/j.biopha.2022.113006](https://doi.org/10.1016/j.biopha.2022.113006) | Hippocampus (rat, translational arm) | – | FoxP3 (IHC) | Western blot HMGB1/RAGE/NF-κB | Dexmedetomidine mechanistic study; Treg analysis limited. |
| Wang F. et al. (2023a) | [PMID:37534273](https://pubmed.ncbi.nlm.nih.gov/37534273/) [doi:10.3389/fneur.2023.1217979](https://doi.org/10.3389/fneur.2023.1217979) | Peripheral blood (human, stroke cohort) | CD4, CD25 | FoxP3 | Cytokine panel | Th17/Treg imbalance correlated with 3-year cognitive decline trajectory. |
| Tian A. et al. (2015) | [PMID:25216867](https://pubmed.ncbi.nlm.nih.gov/25216867/) [doi:10.1007/s10753-014-9956-4](https://doi.org/10.1007/s10753-014-9956-4) | Spleen, blood (mouse, hepatectomy) | CD4, CD25 | FoxP3 | Cytokine panel | Vitamin D modulation of Th17/Treg balance after hepatectomy. |
| Wang Y. et al. (2023b) | [PMID:36800974](https://pubmed.ncbi.nlm.nih.gov/36800974/) [doi:10.1186/s12974-023-02721-0](https://doi.org/10.1186/s12974-023-02721-0) | CNS (mouse LPC demyelination) | CD4, CD25 | FoxP3 | Adoptive transfer; pyroptosis assays | Treg-mediated suppression of microglial pyroptosis via TLR4/MyD88/NF-κB. |

**Supplementary Figure S1. Search-strategy schematic and screening workflow**

Conceptual depiction of the multi-database literature search and screening workflow used for this systematic review (described in Sections S1.2–S1.6).


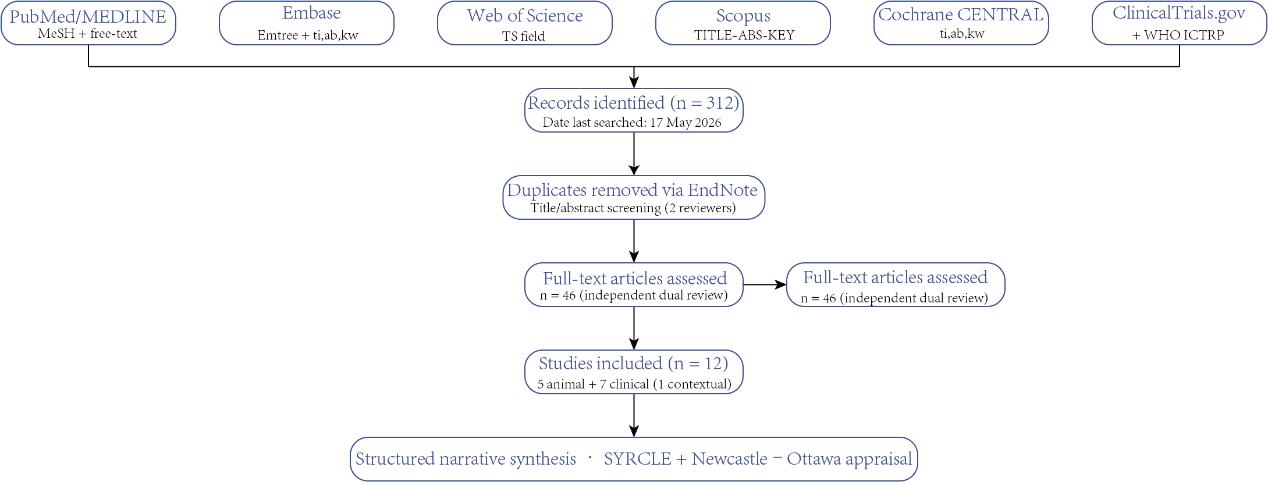

Supplement: Supplementary file 1 [file Data_Sheet_1.DOCX]
